# Multimode Operation of a Superconducting Nanowire Switch in the Nanosecond Regime

**DOI:** 10.1021/acsnano.5c03718

**Published:** 2025-08-04

**Authors:** Zoltán Scherübl, Mátyás Kocsis, Tosson Elalaily, Lőrinc Kupás, Martin Berke, Gergő Fülöp, Thomas Kanne, Karl K. Berggren, Jesper Nygård, Szabolcs Csonka, Péter Makk

**Affiliations:** † Department of Physics, Institute of Physics, Budapest University of Technology and Economics, Műegyetem rkp. 3, Budapest H-1111, Hungary; ‡ MTA-BME Superconducting Nanoelectronics Momentum Research Group, Műegyetem rkp. 3, Budapest H-1111, Hungary; § Low-Temperature Laboratory, Department of Applied Physics, Aalto University School of Science, P.O. Box 15100, Aalto FI-00076, Finland; ∥ Center for Quantum Devices and Nano-Science Center, Niels Bohr Institute, 4321 University of Copenhagen, Universitetsparken 5, Copenhagen DK-2100, Denmark; ⊥ Research Laboratory of Electronics, 2167 Massachusetts Institute of Technology, Cambridge, Massachusetts 02139, United States; # HUN-REN Centre for Energy Research, Konkoly Thege Miklós út 29-33, Budapest H-1121, Hungary; ¶ MTA-BME Correlated van der Waals Structures Momentum Research Group, Műegyetem rkp. 3, Budapest H-1111, Hungary

**Keywords:** gate tunable supercurrent, superconducting electronics, superconductivity, nanowire, switch, fast operation

## Abstract

Superconducting circuits are promising candidates for future computational architectures; however, practical applications require fast operation. Here, we demonstrate fast, gate-based switching of an Al nanowire-based superconducting switch in time-domain experiments. We apply voltage pulses to the gate while monitoring the microwave transmission of the device. Utilizing the usual leakage-based operation, these measurements yield a fast, 1−2 ns switching time to the normal state, possibly limited by the bandwidth of our setup, and a 15−20 ns delay in the normal to superconducting transition. However, having a significant capacitance between the gate and the device allows for a different operation, where the displacement current, induced by the fast gate pulses, drives the transition. The switching from superconducting to the normal state yields a similar fast time scale, while in the opposite direction the switching is significantly faster (4−6 ns) than the leakage-based operation, which may be further improved by a better thermal design. The measured short time scales and the displacement current-based switching operation will be important for future fast and low-power-consumption applications.

## Graphical abstract

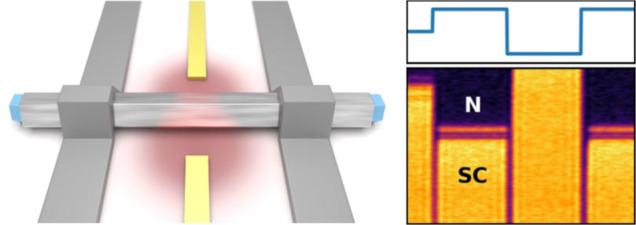

In 2018 De Simoni et al. demonstrated a novel operation of a superconducting circuit, where the switching current of a narrow, fully metallic wire was suppressed and eventually quenched by applying a DC voltage to a nearby gate electrode.^1^ This effect was termed gate-controlled supercurrent (GCS), and since then it has been investigated intensively.^2–35^ For a detailed review of the field see ref 36.

Such an all electrically driven, fully metallic, superconducting switch could be an important building block of superconducting computing structures^36–38^ since the superconducting state promises dissipationless operation, whereas the electrical control suggests a scalable architecture. However, for practical applications, fast switching is required. Up to now three studies have investigated the switching speed and found ∼90 ns,^15^ ∼25 ns,^34^ and ∼40 ns^39^ time scales, which were limited by their setup in all of these cases.

In earlier works the GCS was explained by a purely electric-field-based effect,^1,3,5–8,10,10–12,19,20,29,31^ but later works showed that the underlying mechanism in most cases is related to the leakage current in the substrate at high gate voltages.^13,15–18,24,26,33^ Although the speed of a pure electric field based switching is expected to be only limited by the superconducting gap, as the optical conductivity becomes finite for larger frequencies, yielding up to close to THz operation speed,^36^ a leakage-based switching can be strongly limited by the large resistance of the leakage channel, stray capacitances, and internal thermal time scales. Fast operation of superconducting electronics was already reported in, e.g., rapid single flux quantum^40^ and nanowire cryotron^41^ devices up to 770 GHz and 615 MHz operation speed, respectively.

Aside from the leakage-current-based mechanisms, having a non-negligible capacitance *C* between the gate electrode and one of the contacts (see Figure 1a) allows for a change of the gate voltage to induce a current in the lead, called the displacement current, *I*_disp_ = *C*·d*V*_g_/d*t*, which can switch the device to the normal state if it exceeds the switching current. This displacement-current-based architecture could be used for low-dissipation classical computing, pulse generation in neuromorphic applications,^42–45^ or combined with the leakage current could be operated as a switch in quantum electronic devices.^36–38^

**Figure 1:**
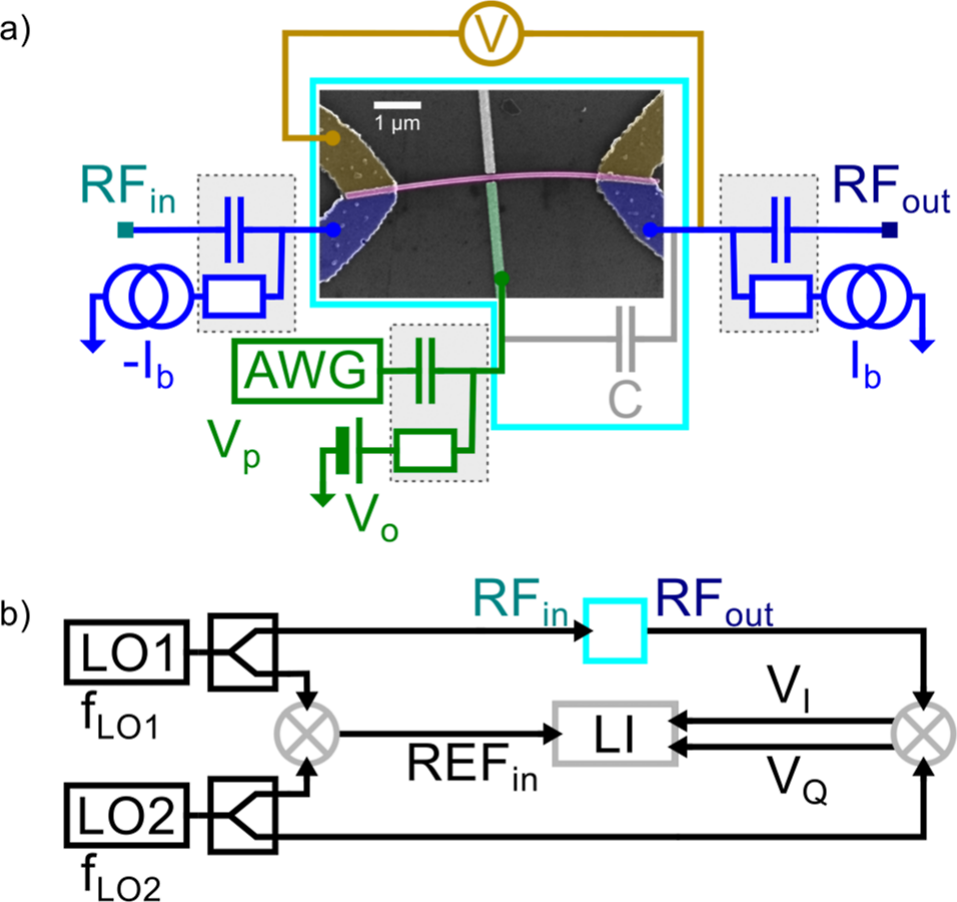
The device and the measurement setup. (a) The false-colored SEM image of the measured device along with the circuit diagram. The epitaxial Al shell of the InAs nanowire (purple) serves as the superconducting wire, contacted in a quasi-four-point geometry. Bias tees (gray rectangles) allow us to combine DC and RF signals applied on the wire. The voltage drop across the wire is measured (yellow) to perform DC characterization. The side gate (green) is also connected to DC and RF sources (AWG, arbitrary waveform generator) to allow for fast pulsing. Due to the design (see the Supporting Information Section I), a substantial capacitance, *C* (in gray), is formed between the gate electrode and ohmic contacts. (b) RF measurement setup. The cyan box corresponds to the same on (a). The effect of the gate pulses was detected as the modulation of the transmission of a *f*_LO1_ = 3.95 GHz signal (generated by a local oscillator, LO1) through the device, which was measured by a lock-in amplifier (LI) after downconversion using another local oscillator, LO2 generating *f*_LO2_, and using IQ (or quadrature) mixers, hence both quadratures (*V*_I_ and *V*_Q_) of the signal were measured.

In this paper, we determine the switching times corresponding to both the leakage- and the displacement-current-driven switching mechanisms by measuring time-resolved RF transmission of a superconducting switch while different pulses were applied on the gate electrode. We show that in both cases the operation is limited by the normal to supra transition, which is 10−20 ns for the leakage-based operation and 4−6 ns with the displacement-current-based operation. The switching to the normal state is 1−2 ns for both mechanisms, presumably limited by the bandwidth of our setup. The former switching is probably limited by thermal time scales in the system, which could be optimized by proper system design.

## Results

Our GCS device is based (see Figure 1a) on an InAs nanowire with an epitaxial Al shell (purple),^17^ where the 20 nm-thin Al shell serves as the superconducting wire. A gate electrode (green) is fabricated 40 nm from the wire by electron beam lithography. The wire is contacted in a quasi-four-point geometry by Ti/Al leads (yellow and blue). The GCS effect in this system was previously demonstrated in refs 17 and 33 . The current leads (blue) are coupled to both RF and DC lines through on-board bias tees (gray dashed rectangles). The device was symmetrically current-biased (i.e., voltages with opposite polarity were applied on the two sides of the wire to generate the current) to keep its potential fixed during biasing. The voltage probe leads (yellow) are connected only to the DC lines. The gate electrode (green) is also connected to a bias tee to combine the DC gate voltage with fast pulses or continuous wave excitations.

To investigate the device response to fast gate operations, we have studied the time dependence of a high-frequency signal transmitted through the device, which was measured by the following way (the RF measurement setup is illustrated in Figure 1b). A fixed *f*_LO1_ = 3.95 GHz signal was applied on one of the current leads of the device and the output signal was measured after downconversion (using IQ mixers) with a second local oscillator (LO2) with frequency *f*_LO2_. Two different measurement schemes were used, technique A (heterodyne): the transmitted signal was downconverted to a few hundred MHz (within the 600 MHz bandwidth of the Zurich Instruments (ZI) UHFLI (UltraHigh Frequency Lock-In), denoted by LI on Figure 1b) and measured by a lock-in technique and technique B (homodyne): the measured signal was downconverted to DC with *f*_LO2_ = *f*_LO1_ and the signal was directly digitized by the UHFLI. With technique A the reference signal was also generated by downconversion, while in technique B it was neglected. Further details of the device and the setup are given in the Methods and Supporting Information Section I.

Figure 2a shows the typical GCS behavior, the reduction of the wire’s switching current with increasing gate voltage, through the four-point voltage drop on the wire, *V*_4p_ as a function of the DC gate voltage, *V*_o_, and the DC bias current, *I*_b_. Up to roughly 14 V the switching current is not affected by the gate voltage, above which the switching current starts to decrease and eventually vanishes at the threshold voltage, *V*_th_ ≈ 19 V. At the same time the leakage current, measured on the gate line, is increasing exponentially (see (c)).^17,26,33^ Figure 2b shows the simultaneously measured RF transmission, as the magnitude of the measured heterodyne voltage, $\left|V_{Het}\right|$ , using technique A at *f*_LO1_ = 3.95 GHz and *f*_LO2_ = 3.55 GHz. The signal is the highest at a low bias and low gate voltage in the superconducting state (SC). Toward higher bias currents and gate voltages, the transmission drops, following the increase of the DC resistance plotted on (a). Further details are given in Supporting Information Section III. Figure 2d shows the zero-gate vertical line cuts of (a and b), further highlighting the good correspondence of the DC voltage curve and the RF transmission. The step-like voltage jumps in the *I*–*V* curve (blue), corresponding to the switching currents of different sections of the device,^17^ appear as kinks in the mostly monotonically decreasing transmission (orange). Altogether, our measurements demonstrate that the RF transmission is also a reliable tool to determine the state of the wire, with a much higher measurement speed compared to DC measurements.

**Figure 2:**
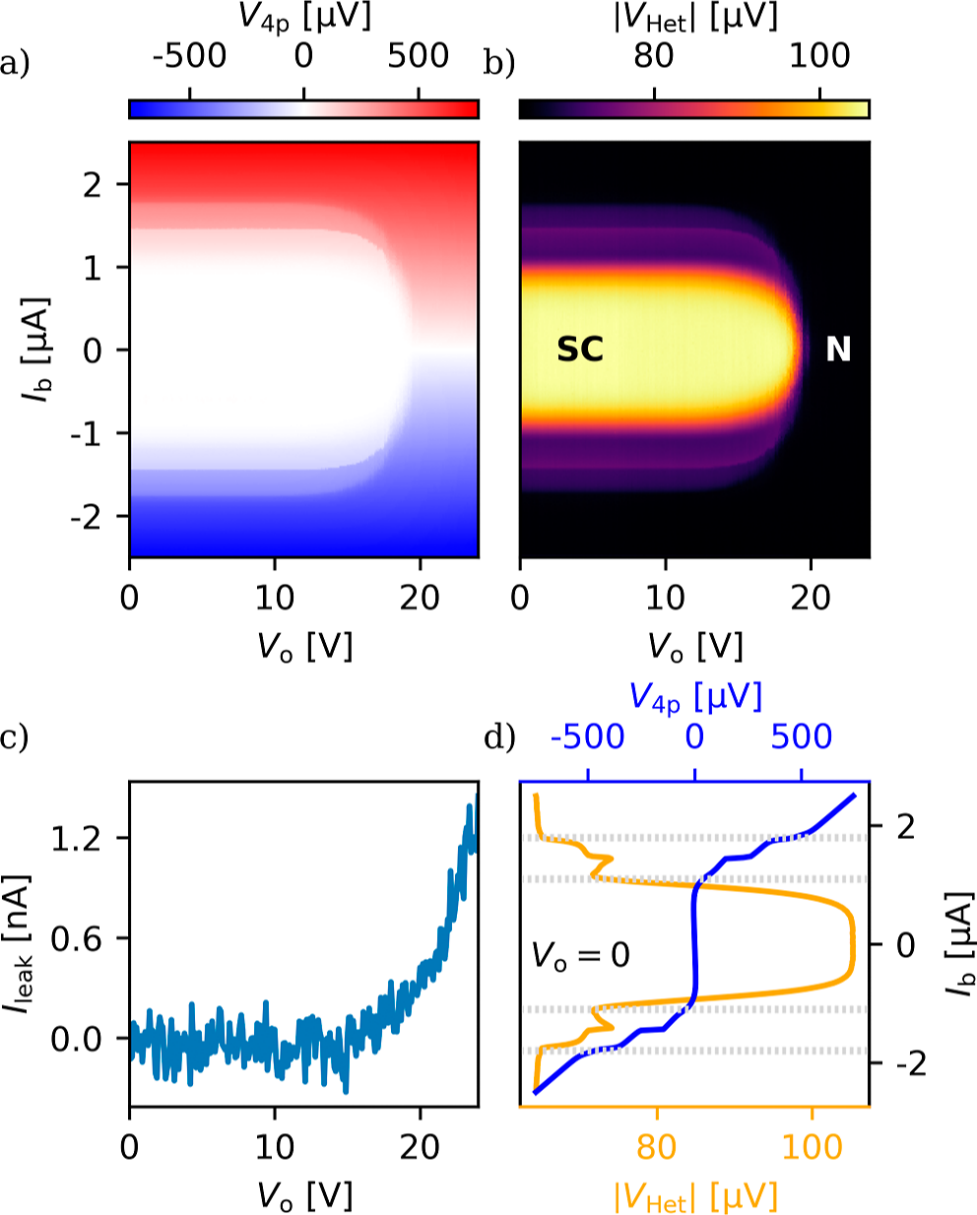
The GCS effect and its RF measurement. (a) The voltage drop, *V*_4p_, on the nanowire as the function of the DC gate voltage and the DC bias current. The switching current starts to decrease at 15 V and vanishes at 19 V. (b) The simultaneously measured transmitted 3.95 GHz RF signal showing a good correlation with the DC measurement. We find high transmission in the superconducting state and low in the normal one. (c) The leakage current in the gate line, also measured simultaneously, increases along with the decreasing switching current. (d) Zero-gate vertical line cuts of (a) and (b), an *I*–*V* curve and the corresponding RF transmission. The gray dashed line is a guide to the eye.

First, we demonstrate the importance of the displacement current in our superconducting switch by investigating the device response to fast gate pulses using the following protocol. A DC offset gate voltage (*V*_o_) was fixed; then a symmetric waveform with zero average, synthesized by an arbitrary waveform generator (ZI HDAWG, denoted by AWG on Figure 1a) was applied on the RF input of the gate, and at the same time the time-resolved RF transmission of the wire was recorded using technique A. To simultaneously have a good enough time resolution and a good signal-to-noise ratio, the outlined sequence is repeated and averaged 500−2000 times, where special care was taken to synchronize the pulses and the data acquisition. These measurements were carried out at zero DC current bias.

Two of such measurements are presented in Figure 3a,b, where the top row shows the applied waveform, *V*_p_, the bottom row shows the measured transmission, the magnitude of the heterodyne voltage, $\left|V_{Het}\right|$ as a function of time and the DC offset gate voltage, *V*_o_. On (a), a measurement is shown where a ±2.5 V trapezoid-like wave was applied on the gate, with a slow, 200 ns ramp time (i.e., 25 V/μs ramp rate). Taking a vertical cut of the measurements close to *t* = 0 one obtains the suppression of superconductivity with DC gate voltage, already presented in Figure 2a,b: with increasing gate voltage the wire switches from an SC state (yellow) to normal (N) (purple—black). The threshold voltage differs from the one in Figure 1 due to the so-called training effect (see the Methods).^17^ At lower gate voltages, below 20 V, the wire remains in the SC state throughout the pulse sequence since the total gate voltage (*V*_g_ = *V*_o_ + *V*_p_) never exceeds the threshold voltage. However, for larger DC gate voltages, e.g., 21 V, a change in transmission is visible for the positive half of the pulse, where the total gate voltage (23.5 V) exceeds *V*_th_ and hence the superconductivity is quenched (black region). On the negative side of the pulse the device switches back to the SC state (yellow). These results demonstrate that the state of the wire follows the applied pulse sequence and it is only determined by the total gate voltage. This suggests that the wire state changes much faster than the ramp time of the pulse sequence and the device behaves in a quasi-stationary way.

**Figure 3:**
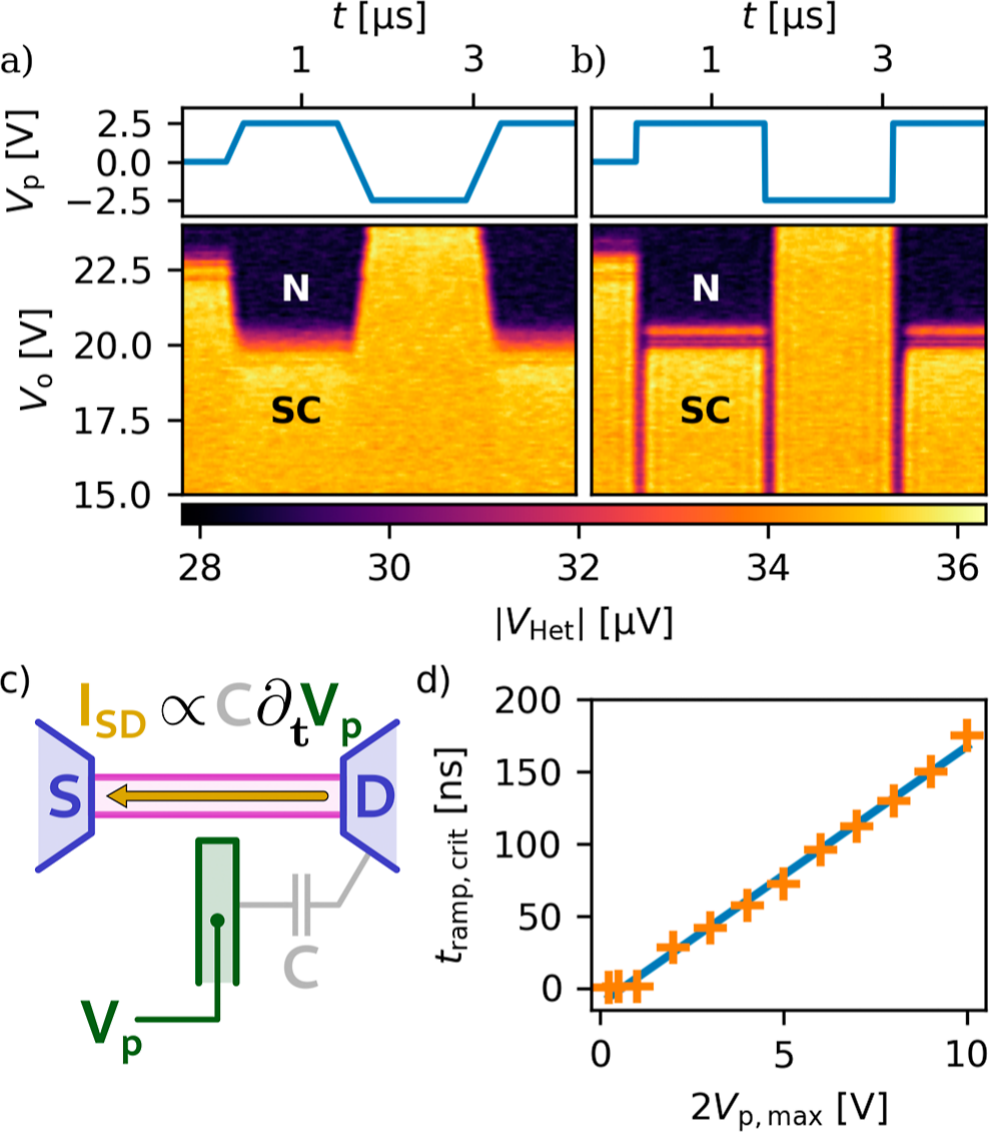
Pulsed measurements (a) and (b). Top panel: the applied pulse sequence, *V*_p_, (a) ramped, trapezoid-like pulse, (b) square pulse. Bottom panels: the measured transmission (using technique A) as a function of time and the DC offset gate voltage. (a) In case of a slowly ramped pulse the transmission follows well the total gate voltage, *V*_0_ + *V*_p_, (b) with the square pulse additional normal regions appear (vertical purple lines) each time the gate voltage suddenly changes, due to the generated displacement current and the resulting switching. (c) Origin of the displacement current, being proportional to the capacitance between the gate and the device, and gate voltage changing rate, (d) the critical ramp times (see text for definition), which are needed for the switching, show a clear linear trend with the pulse amplitude, in agreement with the displacement current picture.

Figure 3b shows a similar measurement, however, using a square pulse (with ramp times limited by our instruments, ∼1 ns). Compared to (a), the boundaries between the SC and N regions become vertical, but the apparent threshold voltage again shifts with −*V*_p_ as in the previous case. In addition, purple vertical lines appear at both the rising and falling edges of the pulse. These features extend down to zero DC gate voltage (not shown here), but they do not extend into the normal region. Therefore, this effect has to be related to the state of the wire. This surprising feature means that independently from the DC gate voltage, the wire switches to the N state each time the gate voltage is rapidly changed. After the rapid gate ramp, the wire does not remain in the N state, but it switches back to the SC state if the total voltage does not exceed the threshold voltage. We attribute this switching effect to the displacement current in the wire and the leads, induced by a change of the gate voltage. Though the capacitance between the gate and the wire is rather small (30 aF), a substantial capacitance of 10−100 fF forms between the leads and the gate line on the millimeter-scale wiring segments from the device to the bonding pads (see the schematic in Figure 3c and Supporting Information Section I for the detailed discussion). Due to the asymmetric design of the device, such that the gate has a significantly larger capacitance to the right contacts than to the left ones, the displacement current results in a net current across the wire. If the induced displacement current is larger than the switching current, the wire indeed switches to the N state in response to the fast change of the gate voltage. Since the displacement current is generated by the changing gate voltage, after the ramping period the displacement current decays and the wire switches back to the SC state. Therefore, we call this effect displacement-current-induced switching (DCIS).

Next, we investigated the effect of the ramping speed of the pulse on the DCIS. We repeated the measurement sequence with different pulse amplitudes and ramp times at zero DC gate voltage but with improved time resolution using technique B. We have already shown that if the ramp is slow enough, the DCIS is absent, so for a given pulse amplitude one can find a ramp time, below which the displacement current is large enough to switch the wire, but not above. The obtained critical ramp times, *t*_ramp,crit_ are shown in Figure 3d as the function the gate pulse amplitude. Details of the evaluation are given in the Supporting Information Section V. The points indicate a clear linear trend, consistent with the displacement current picture, i.e., *I*_disp_ = *C*_eff_·d*V*_g_/d*t*, where *C*_eff_ is the effective capacitance between the gate and the device. From a linear fit, in blue, assuming that the displacement current is equal to the lowest switching current, *I*_disp_ = *I*_c1_ = 1.1 μA, we found *C*_eff_ = 20 fF, which is comparable with our finite element simulation (see Supporting Information Section I for the details with a short discussion on the possible errors as well).

After these time-domain measurements, we have turned to frequency-domain studies, where the response to a harmonic excitation can be addressed. Applying a continuous sine wave on the gate opens up the possibility of studying mixing effects. The periodic modulation of the gate voltage also modulates the high-frequency transmission (by changing the state of the wire) and mixes the signal on the gate with the readout tone. To investigate this effect the output signal was mixed down to 133 MHz (technique A with *f*_LO1_ = 3.95 GHz and *f*_LO2_ = 3.817 GHz) and an FFT was taken (using the scope module of the UHFLI). Two of such examples are shown in Figure 4a,b, where the FFT amplitude is shown as a function of the offset gate voltage and the frequency. During these measurements a combined DC plus AC voltage was applied on the gate: $V_{o}+A_{g}\sin\left(2\pi f_{g}t\right)$ , with *A*_g_ = 0.5 V for (a) and 2 V for (b) with *f*_g_ = 10 MHz. For both measurements, a large signal is visible at *f*_LO1_ − *f*_LO2_ = 133 MHz in the full offset gate voltage range, coming from the measurement tone. For the smaller amplitude on (a) other features show up in a narrow offset gate range from 14 to 16 V at higher and lower frequencies than the measurement tone, marked by the green arrows. These side peaks originate from the mixing of the harmonic gate drive with the transmitted signal. The two dominant sideband peaks that are spaced ±*f*_g_ from the main peak at 123 and 143 MHz are indicated by the arrows. We interpret the presence of these peaks as leakage-driven switching. When the DC offset plus the drive is larger than the threshold, the wire is switched to the N state, but when the total gate voltage decreases below the threshold, the wire switches back to the SC state. Hence, the switching occurs once every period and the transmission is modulated at *f*_g_.

**Figure 4:**
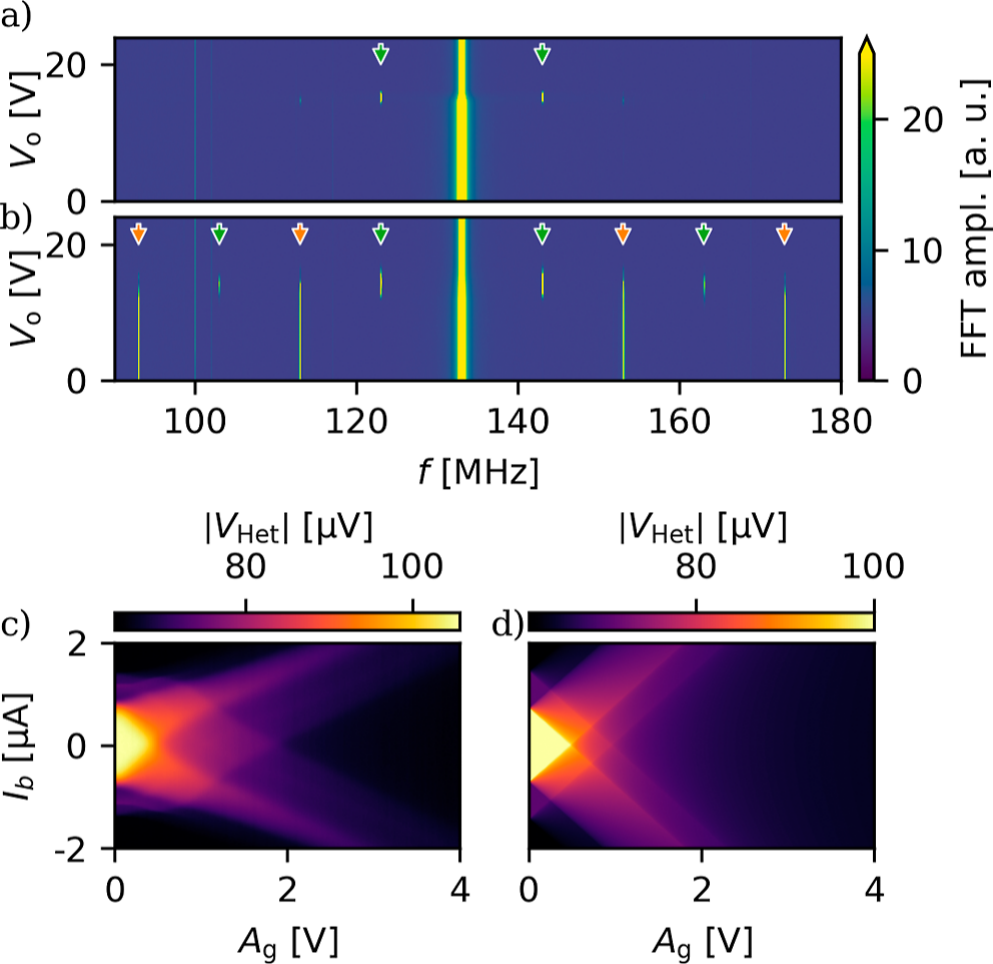
Measurements with a continuous harmonic drive. (a) and (b) FFT spectra of the measured RF signal (after downconversion), while continuously driving the gate at *f*_g_ = 10 MHz, with (a) *A*_g_ = 0.5 V, (b) *A*_g_ = 2 V amplitude as the function of the DC offset gate voltage, measured at a zero DC bias current. The total gate voltage is $V_{g}=V_{o}+A_{g}\sin\left(2\pi f_{g}t\right)$ . The central peak at 133 MHz corresponds to the direct transmission of the readout signal. The sideband peaks are the result of mixing *f*_g_ with the readout signal. The odd sideband peaks at 123 and 143 MHz around *V*_o_ = 15 V, the threshold voltage, are due to the gating effect on (a), while the even sideband peaks at 113 and 153 MHz, extending down to zero gate voltage on (b), are the result of DCIS. (c) Time-averaged transmission of the device with an *f*_g_ = 15 MHz harmonic drive as the function of the drive amplitude, *A*_g_, and the DC bias current, *I*_b_, at a zero DC gate voltage. The superconducting region (yellow) shrinks with increasing drive amplitude. At large amplitudes better transmitting branches appear at a high bias, where the displacement current is partially compensated by the DC bias. (d) Simulation for (c), where the instantaneous transmission is determined by the total current flowing through the device (the sum of the DC bias and the harmonic displacement current) and time-averaged for one period of the drive, using *I*_c1_ = 0.9 μA, *I*_c2_ = 1.8 μA, *C* = 20 fF.

In sharp contrast, for *A*_g_ = 2.5 V, four pairs of sideband peaks appear around the main peak (see (b)). The first and third sideband peaks (indicated by green arrows) appear only around the threshold gate voltage, while the second and fourth (orange arrows) extend down to the zero DC gate voltage. The latter ones appear due to the displacement current since for both increasing and decreasing gate voltages the displacement current exceeds the switching current. Therefore, the wire switches to the N state *twice* in one period of the gate drive, resulting in a frequency doubling. These measurements provide a clear distinction between the leakage-driven and displacement-current-based switching mechanisms. As a control experiment, at large offset gate voltage, in the N state, no sideband could be observed. We also measured the mixing effects at several drive frequencies and amplitudes. Using a simple model, we could well reproduce the general tendencies as we demonstrate in Supporting Information Sections VII−VIII.

The DCIS picture can be further validated by applying a DC bias current that adds to the current generated by the harmonic drive on the gate. For this, we have applied a few-MHz sine tone on the gate with varying amplitudes and measured the time-averaged transmission (using technique A) of the wire as a function of the drive amplitude, *A*_g_, and the DC bias current, *I*_b_, at zero offset gate voltage (time-resolved measurements will be discussed later). An example is shown in Figure 4c with an *f*_g_ of 15 MHz. At small drive amplitudes, the features resemble well the undriven case, i.e., the curve presented in Figure 2d with a high transmission at a low DC current, and with decreasing transmission as the device switches to the N state in several steps with increasing bias current. At higher drive amplitudes, the bright yellow, fully SC region shrinks and vanishes around an *A*_g_ ≈ 0.5 V amplitude. At the same time two better-transmitting branches develop diagonally. At large amplitudes, above *A*_g_ = 2 V, close to zero DC current the transmission is close to the value of the normal case, but surprisingly, by applying a finite DC bias current, one finds higher transmissions in the branches.

The main features of this measurement can be captured using a simple circuit model (see results in Figure 4d), where the resistance of the wire depends on the total current flowing through it and increases in a step-like fashion. The current is the sum of the DC bias and the AC displacement current component; therefore, the resistance becomes time-dependent. The instantaneous transmission is calculated from the resistance with a phenomenological formula. Finally, time-averaging for one period gives the average transmission shown in Figure 4d, reproducing well the experimental data. Details of the model are described in the Methods. For the SC triangle at small drive amplitudes and DC bias, the total current is always below the lowest switching current, *I*_c1_. At the tip of the triangle, the amplitude of the displacement current reaches *I*_c1_. This feature allows for an alternative way to determine *C*_eff_, which is presented in Supporting Information Section VI, yielding a good agreement with the one extracted from pulsed measurements in Figure 3d. The diagonal, high-bias features for large amplitudes originate from the partial compensation of the displacement current with the bias current.

To study the time scales of the leakage-based switching mechanism close to the threshold gate voltage, we return to the pulsed measurements (technique B). We suppress the DCIS by choosing a slow enough ramping of the gate voltage such that the displacement current does not switch the wire. Then close to the threshold gate voltage, we evaluate the delay of the response of the wire compared to the arrival of the gate pulse.

Such a measurement is presented in Figure 5 using a similar ramped pulse sequence as in Figure 3a with a 2.5 V amplitude and 100 ns ramp time. We focus on the ramp up and ramp down part of the pulse sequence. As the total gate voltage is given by the sum of the DC offset and the pulse, in the ramp up phase of the pulse, the DC gate threshold is decreasing. The sudden change in the signal at *t* = 2.05 and 1.65 μs marks the point in time when the gate pulse reaches the wire and the shifting of the threshold starts. Using this, we can calculate, based on the total gate voltage, the actual DC threshold voltage where the SC to N transition should take place at a given time. We indicate this threshold by the blue dashed line. The white points show the position of the transition evaluated from the high-frequency transmission measurements (yellow to purple color change, for the detailed description of the evaluation see Supporting Information Section IX). For the ramp-up phase (left panel), the transition to the N state roughly coincides with the blue line. On the contrary, for the down ramp (middle panel) there is a clear delay in the signal with respect to the blue dashed line. This indicates that the switching to the N state by the leakage current is almost instantaneous, but the switching back to SC is delayed. To quantify the time scales, for each time trace we identify the time delay between the expected and measured transition times and created a histogram out of the time delays (see (c)). For the ramp up, the mean value is less than 1 ns, while for the down ramp it is 19 ns. The spread of the distribution can also originate from the noise of the readout signal and the jitter of the pulse. Our measurements indicate that the leakage current itself responds quite quickly to the change in the gate voltage, the wire switches to N within a few nanoseconds, but the switching back to the SC state is limited, presumably due to thermal effects. The ns-switching time is one order of magnitude faster than the previously reported switching speed.^34^ As soon as the wire switches to the N state, dissipation occurs, and due to the low phonon cooling efficiency at low temperatures, the switching back is slow.

**Figure 5:**
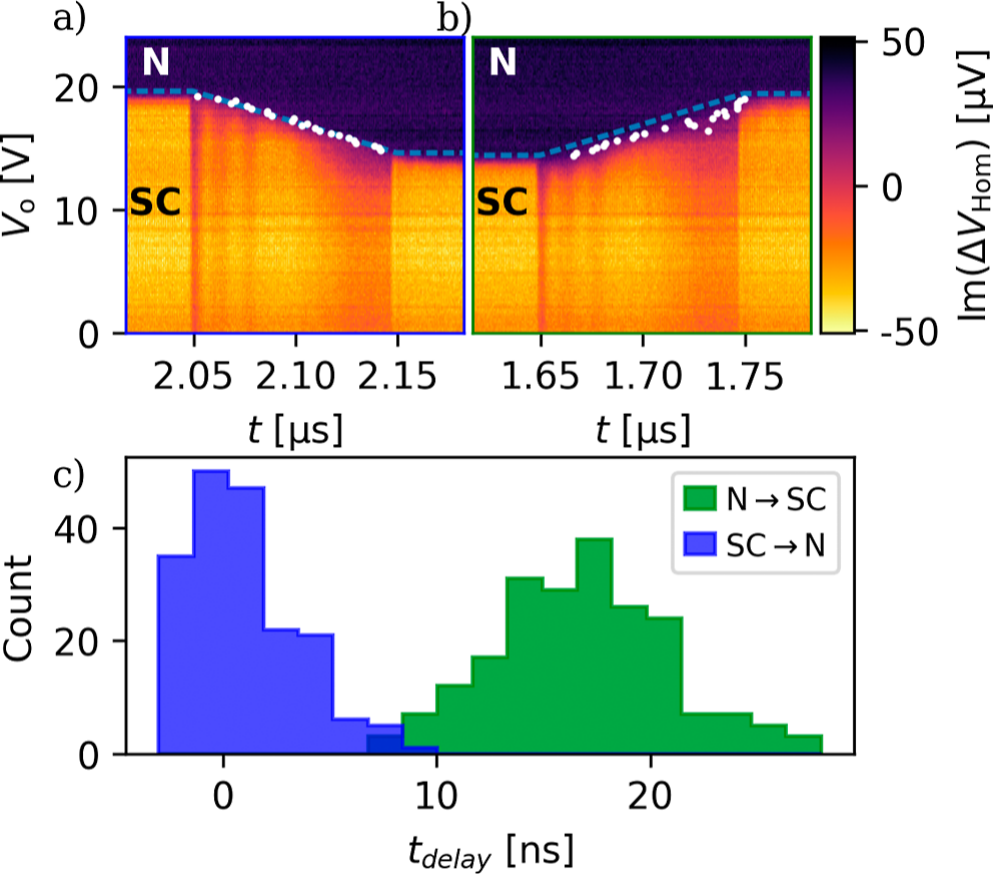
Time scales of the leakage-based switching. (a) and (b) Zoom-in to the rising and the falling edge of the time dependence of the RF readout signal using ramped, trapezoid-like gate pulses, with a 100 ns ramp time and a 2.5 V pulse amplitude. Blue lines mark the *V*_o_ value where the offset gate voltage plus the pulse is equivalent to the threshold voltage. White dots indicate the point in each time trace where the signal reaches the level corresponding to the normal state (purple) or starts to decay from that level. The horizontal separation of the blue lines and the white dots corresponds to the delay times of the leakage-based switchings and plotted on (c) as a histogram for 9 up and down steps. The switching to the normal state happens in a few nanoseconds (blue), but the normal to supra transition is delayed by about 15−20 ns (green).

Finally, we quantify the time scales corresponding to the DCIS. For this, we used a harmonic driving on the gate and measured the time-resolved transmission (using technique B) as the function of the DC bias current, *I*_b_, at a zero DC offset gate voltage. An example is shown in Figure 6a with *f*_g_ = 10 MHz and *A*_g_ = 5 V. The measurement indicates that the borders of the different regions (yellow for the SC and purple for the N state) oscillate sinusoidally with the applied gate drive. This is because at each time the sinusoidal displacement current can be compensated by the DC current, providing a zero total current, and hence, the wire is in the SC state. When it happens at the zero displacement current (extrema of the driving signal), the SC region is centered around the zero DC current, whereas when the displacement current is finite, the SC region is shifted vertically. These features are nicely captured by our model (see Figure 6b). In the model (detailed in the Methods), the state of the wire is only determined by the total current, which is the sum of the DC bias and the induced sinusoidal displacement current.

**Figure 6:**
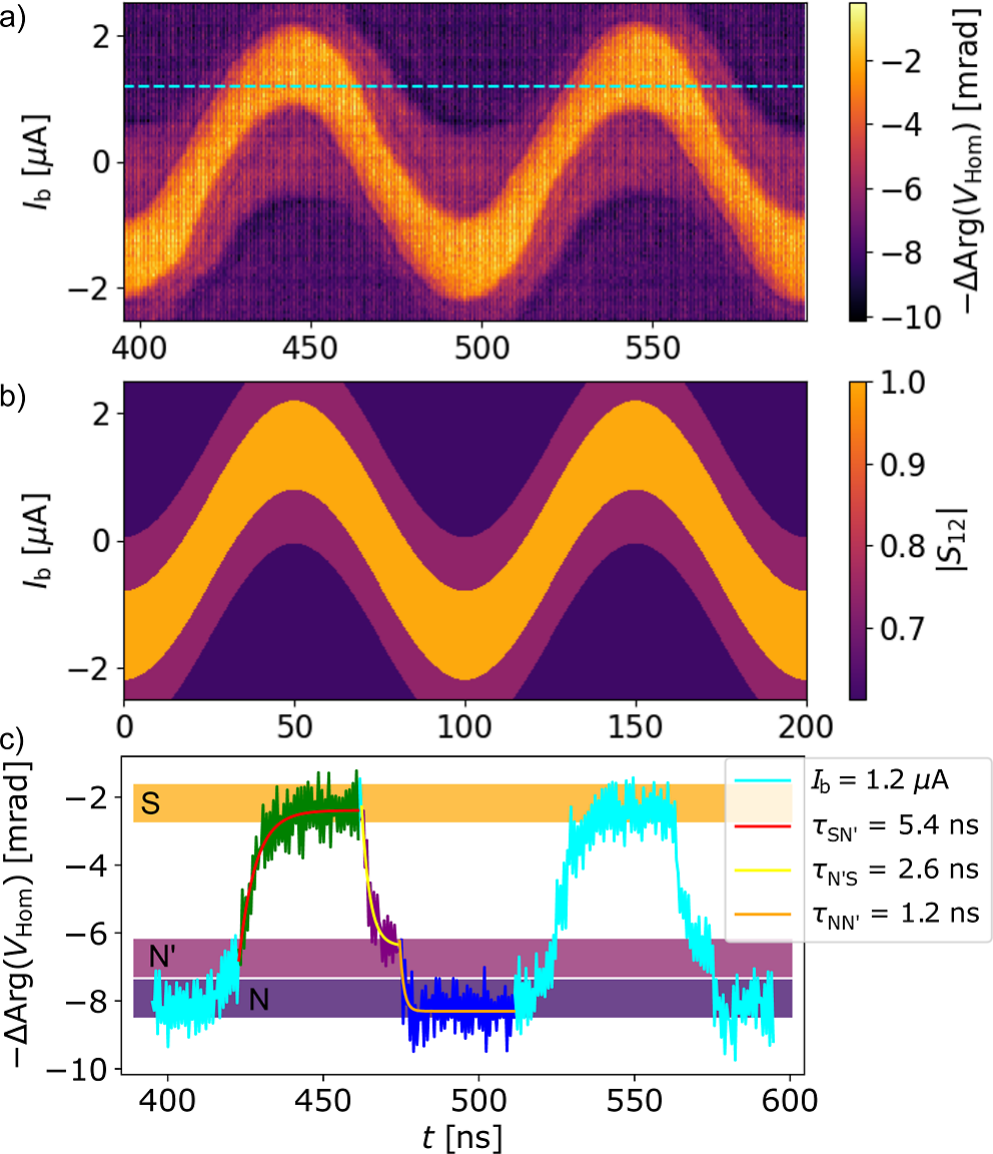
Time scales of the displacement-current-based switching. (a) Time-resolved transmission (phase of the homodyne voltage) as the function of the DC bias current at a zero DC gate voltage under harmonic drive using *f*_g_ = 10 MHz and *A*_g_ = 5 V. The boundaries of the different regions shift along the *I*_b_ axis with the driving signal. (b) Simulation of the measurement of (a). (c) Exemplary horizontal cut from (a) at the cyan line; the color-coded sections are fitted by exponential functions yielding a 5−6 ns switching time from N′ to S and 1–2.5 ns for S to N′ and N′ to N.

To evaluate the time scales we focus on an exemplary time trace of (a) taken at *I*_b_ = 1.2 μA, which is plotted on (c). The signal changes periodically between three signal levels, yellow as S, purple as N′, and dark purple as N. As noted before, our device turns to normal in two steps.^17^ The transitions between these states, visible in (c), are fitted with exponential functions with the time constants associated with the time scales of the DCIS. Due to the low resolution, we did not fit the N → N′ transition. The N′ → S transition (green section) yields a time scale of τ_SN′_ ≈ 5.4 ns. When fitting several of these processes, we obtain 3.9 ± 1.3 ns independent of the bias current (see additional examples in Supporting Information Section XI). This is significantly faster than the leakage-based switching, which we believe can be explained by the absence of non-equilibrium phonons. With DCIS only quasiparticles are excited in the nanowire, while the leakage current generates non-equilibrium phonons as well, which also have to decay. The other switchings, the S → N′ (τ_N′S_, purple) and N′ → N (τ_NN′_, blue), yield 2.8 ± 0.8 and 1.4 ± 0.5 ns on average (on the particular plotted curves 2.6 and 1.2 ns), respectively, which is in the range of the instrumental limitations of our setup. The fast switching is also demonstrated by pulsed measurements, which are discussed in Supporting Information Section IV.

## Conclusion

In this paper we have studied the time scales associated with the gate-based operation of an Al nanowire superconducting switch by monitoring the transmission of a ∼4 GHz continuous tone through the device. The significant capacitance between the gate electrode and the current leads allows for a new type of operation, where the gate pulse switches the wire to a normal state via inducing a displacement current in the wire. The switching to the normal state is close to our instrumental limits and happens in about 1−3 ns, which is more than one order of magnitude faster than previously reported values.^15,34^ The switching from the normal state back to the superconducting state is somewhat slower, 4−6 ns, presumably limited by the relaxation of the quasiparticles.

By suppressing the DCIS mechanism, we also explored the time scales associated with the leakage-based switching, giving a similarly fast switching to the normal state, but a slower, 15−20 ns back to the superconducting state. The latter is presumably limited by the combined thermal relaxation of the leakage-current-generated phonons and quasiparticles in the superconductor.

These time scales suggest that the displacement-current-based operation is more promising for an application than the leakage-based operation. In the DCIS operation using square pulses the device switches only during the rise- or fall-time of the pulse, which could be used for fast pulse generation, e.g., in neuromorphic architectures.^42–45^ Moreover, a continuous (harmonic) driving can keep the device in a normal state arbitrarily long. For scaling up these devices, the operation should be local: the application of the gate voltage should address only a single nanowire. However, for the DCIS mechanism this can be well engineered by well-defined capacitors, for the leakage-based mechanism this could be a challenge for some substrates.^24^ The footprint of a single device could be significantly decreased by using well-designed capacitors, with a high dielectric constant (e.g., a plate capacitor with *A* = 1 μm^2^ area assuming ε_r_ = 25 dielectric constant of HfO_2_ with 10 nm thickness yields 22 fF, similar to the one in our device), whereas the crosstalk can be mitigated by using ground planes to screen the unwanted stray fields. At the moment the operation speed is limited by the relaxation, which may be improved by better thermalizing the device or by using, e.g., quasiparticle traps. Finally, for finite gate voltages, the two operation methods can be combined, where the fast switching is obtained by the DCIS mechanism, whereas the long-term memory of the state could be provided by the leakage current. Or alternatively, one can use devices, with sizable switching-retrapping current asymmetry, where using a current bias set point between the two values could enable DCIS-based S to N switching by a positive pulse, while N to S switching is induced by a negative pulse.

A possible application utilizing the DCIS could be a microwave comparator or discriminator, where the sensed AC signal is applied on the gate. If it is strong enough to switch the wire, then a digital output is generated by the state change of the wire. Such an amplifier could find use as a transducer for classical digital signals applied to the cryogenic system for the control of sensors or quantum information hardware. To quantify the possible advantages of this approach to this problem, a more detailed study is required, which is beyond the scope of this paper.

Our device geometry is related to previous approaches such as the nanowire cryotrons,^46^ where fast operation was already demonstrated^41^ at the power dissipation of few tens of nanowatts. Due to the similarities, we expect comparable power dissipation and jitter for our device. Estimates are given in Supporting Information Section XII.

## Methods

### Device and Setup

The investigated device was fabricated by depositing an InAs nanowire with a 20 nm-thick full Al shell layer on an undoped Si wafer with a 290 nm-thick oxide layer. Following nanowire deposition, four Ti/Al contacts with thicknesses of 10/80 nm and two opposite side gates made of Ti/Au with thicknesses of 7/33 nm were patterned using two separate electron beam lithography steps. In the device shown in Figure 1a, the length of the nanowire segment under investigation is approximately 4.2 μm.

The device was contacted in a quasi-four-point geometry. Two leads were connected to both DC and RF lines through on-board bias tees (100 Ω and 10 pF); these were used as biased leads. One of the rest of the contacts was not working, so the DC resistance was measured in a three-point configuration, and the data were later corrected for line resistance of 150 Ω. The presented data correspond to the corrected values. The device was current-biased symmetrically to keep the potential of the wire constant. The RF measurement was carried out by transmitting a 3.95 GHz signal through the device applied to the current leads. The transmitted signal was downconverted by an IQ mixer using a second local oscillator and was measured by a Zurich Instruments UHFLI. Two different measurement schemes were used throughout the paper. First, as per the heterodyne technique A, the transmitted signal was downconverted to a few hundred MHz and was measured by a lock-in technique, where the reference signal was also generated by downconversion. In this case the time resolution was limited by the integration time of the UFHLI, which has a lower limit of 30 ns, yielding ∼100 ns time resolution. Second, as per the homodyne technique B, the signal was directly downconverted to DC and was measured by the scope module of the UHFLI, allowing for a 1.8 GS/s sampling rate, and the time resolution was limited by the 600 MHz bandwidth of the UHFLI. As IQ mixers were used, both quadratures of the signal were measured with both techniques. In case of technique A, the signals at the lock-in inputs are expressed as

1 $$\begin{array}{l} V_{I}\left(t\right)=\left|V_{Het}\left(t\right)\right|\cos\left(\omega t+\mathrm{Arg}\left(V_{Het}\left(t\right)\right)\right)\\ V_{Q}\left(t\right)=\left|V_{Het}\left(t\right)\right|\sin\left(\omega t+\mathrm{Arg}\left(V_{Het}\left(t\right)\right)\right), \end{array}$$

Hence both quadratures contain both the magnitude and the phase. However, for technique B they are

2 $$\begin{array}{l} V_{I}\left(t\right)=\left|V_{\mathrm{Hom}}\left(t\right)\right|\cos\left(\mathrm{Arg}\left(V_{\mathrm{Hom}}\left(t\right)\right)\right)\\ V_{Q}\left(t\right)=\left|V_{\mathrm{Hom}}\left(t\right)\right|\sin\left(\mathrm{Arg}\left(V_{\mathrm{Hom}}\left(t\right)\right)\right), \end{array}$$

i.e., the magnitude and the phase can be determined if both quadratures are measured. All measurements were carried out at 5 to 15 dBm readout power at the top of the cryostat, and the input line had 80 dB nominal attenuation (see Supporting Information Section I).

### Training Effect

Through the measurements the threshold voltage, i.e., the offset gate voltage, where the superconductivity is quenched decreased monotonically. This is due to the so-called training effect,^17^ that is, first a large gate voltage is needed to start the leakage current, but after that the current itself builds a better and better conducting channel, so a lower and lower gate voltage is enough to provide the same leakage current until it saturates. The DC-gated measurements here were carried out in the following chronological order: (i) Figure 3, with *V*_th_ ≈ 23 V, (ii) Figure 2, with *V*_th_ ≈ 19 V, (iii) Figure 5, with *V*_th_ ≈ 17 V and (iv) Figure 4a,b, with *V*_th_ ≈ 15 V.

### Modeling

Here we outline how the effect of the displacement current induced by the harmonic drive on the gate was simulated to generate Figures 4d and 6b. First, we assume two different critical currents, *I*_c1_ and *I*_c2_, and different constant resistance values above each critical current, *R*_1_ and *R*_2_, corresponding to the N′ and N states, respectively. This captures the effect of having more than one step. Second, we assume that the resistance jumps at *I*_ci_, but otherwise it is constant, therefore

3 $$R\left(I\right)=\left\{ \begin{array}{ll} 0 & \mathrm{if}\;I<I_{c1}\\ R_{1} & \mathrm{if}\;I_{c1}\leq I<I_{c2}\\ R_{2} & \mathrm{if}\;I_{c2}\leq I \end{array}\right.$$

The current flowing through the wire is the sum of a DC component and an AC one, coming from the biasing and displacement current, respectively, hence

4 $$I\left(t\right)=I_{b}+I_{AC}\;\sin(\omega t).$$

The AC current can also be expressed as

5 $$I_{AC}\left(t\right)=I_{disp}=C\frac{dV_{g}\left(t\right)}{dt}=C\omega V_{g}\;\sin(\omega t).$$

Due to the displacement current, the resistance also becomes time-dependent. In the model, we used *C* = 20 fF.

Finally, we assume that the transmission instantaneously follows the resistance

6 $$\left|S_{12}\right|\left(t\right)=\frac{474\Omega }{474\Omega +R\left(t\right)},$$

where the phenomenological formula introduced in Supporting Information Section III is used. These steps yield the time-dependent plot in Figure 6b. To generate Figure 4d the transmission is time-averaged

7 $$\left|S_{12}\right|=\left\langle \left|S_{12}\right|\left(t\right)\right\rangle$$

where ⟨·⟩ denotes the averaging for one period of the gate signal.

The parameters used to generate the two figures are.

 
*C*_eff_

*R*_1_

*R*_2_

*I*_c1_

*I*_c2_
Figure 4d20 fF200 Ω300 Ω0.9 μA1.8 μAFigure [Fig fig6]b20 fF170 Ω300 Ω0.7 μA1.55 μA

## Supplementary Material



## Data Availability

The data that support the plots within this paper and other findings of this study are available online: 10.5281/zenodo.15775219.

## Notes

Details of the setup and further measurements and simulations (PDF)
