# Correction to “Multimode Operation of a Superconducting Nanowire Switch in the Nanosecond Regime”

**DOI:** 10.1021/acsnano.5c18095

**Published:** 2025-10-31

**Authors:** Zoltán Scherübl, Mátyás Kocsis, Tosson Elalaily, Lőrinc Kupás, Martin Berke, Gergő Fülöp, Thomas Kanne, Karl Berggren, Jesper Nygård, Szabolcs Csonka, Péter Makk

In our original article, the shared equal contribution of the first and second author was not marked; moreover, the author contribution was incomplete. Here, we give the corrected author contribution section.

T.E. and M.B. fabricated the device. M.K. developed the measurement setup. Z.S. and L.K. performed the measurements with the help of M.K. T.K. and J.N. developed the nanowires. The data analysis was performed by M.K., Z.S., and L.K. Modeling and simulation were done by M.K., Z.S., L.K., and G.F. with inputs from M.B. and K.B. The project was guided by Z.S., P.M., and S.C. The manuscript has been prepared by Z.S., M.K., and P.M. with input from all authors.

