# Supplementary material for: Multimode Operation of a Superconducting Nanowire Switch in the Nanosecond Regime
Source: ACS Nano. 2025 Aug 4;19(32):29207–15. doi: 10.1021/acsnano.5c03718 (PMC12368992; doi:10.1021/acsnano.5c03718)
Supplement: Supplementary file 1 [file nn5c03718_si_001.pdf]

Supporting Information for Multimode operation of a superconducting nanowire switch in the nanosecond regime

Zoltán Scherübl,[1,2] Mátyás Kocsis,[1,2] Tosson Elalaily,[1,2,3] Lőrinc Kupás,[1,2] Martin Berke,[1,2] Gergő Fülöp,[1,2] Thomas Kanne,[4] Karl Berggren,[5] Jesper Nygård,[4] Szabolcs Csonka,[1,2,6,*] and Péter Makk[1,7,†]

[1] *Department of Physics, Institute of Physics, Budapest University of Technology and Economics, Műegyetem rkp. 3., H-1111 Budapest, Hungary*

[2] *MTA-BME Superconducting Nanoelectronics Momentum Research Group, Műegyetem rkp. 3., H-1111 Budapest, Hungary*

[3] *Low-Temperature Laboratory, Department of Applied Physics, Aalto University School of Science, P.O. Box 15100, FI-00076, Aalto, Finland*

[4] *Center for Quantum Devices and Nano-Science Center, Niels Bohr Institute, University of Copenhagen, Universitetsparken 5, DK-2100, Copenhagen, Denmark*

[5] *Research Laboratory of Electronics, Massachusetts Institute of Technology, Cambridge, Massachusetts 02139, USA*

[6] *HUN-REN Centre for Energy Research, Hungary, H-1121 Budapest, Konkoly Thege Miklós út 29-33.*

[7] *MTA-BME Correlated van der Waals Structures Momentum Research Group, Műegyetem rkp. 3., H-1111 Budapest, Hungary*

(Dated: August 1, 2025)

I. SAMPLE GEOMETRY AND CROSS-CAPACITANCES

Supp. Fig. 1a shows the exact sample geometry. The device was fabricated on an undoped silicon wafer, covered by 290 nm thermal oxide. The InAs core of the wire is covered by 20 nm in-situ grown epitaxial aluminum layer, the total diameter is about 100 nm. The wire is contacted in quasi-four-point geometry by Ti/Al leads. The 200 nm wide, Ti/Al side gate electrodes are placed about 40 and 60 nm away from the wire. In the measurement only the closer one (G) was connected and used, G' not. The voltage probe lead, V1 was not working during the presented measurements, hence the three-point voltage was measured and later corrected for the line resistance. In this paper only the corrected values are presented as $V_{4\mathrm{p}}$. The predefined Ti/Au wires (like G and I1 on panel a), running close, 20–100 µm far from each other on the length ~ 1 mm yield significant capacitance (see below).

Supp. Fig. 1b shows the schematics of the electrical circuit. The on-board bias tees are made by SMD resistors and capacitors, with 100 Ω and 10 pF for the biased lines and 220 kΩ and 10 pF for the gate electrode. The device was symmetrically current biased by using 100 kΩ resistors at room temperature and voltages with opposite sign on the two resistors. The voltage drop on the device was amplified by a differential amplifier (Basel Precision Instruments SP1004) and measured by an Agilent 34401A multimeter. The leakage current was measured as the voltage drop on a 10 MΩ resistor also at room temperature and another 10 MΩ was used to suppress the noise coming from the multimeter (Agilent 34401A). The pulses and the harmonic drive applied on the gate was synthesized by a Zurich Instruments HDAWG (except for Supp. Fig. 6c and Supp. Fig. 8 where a Rohde&Schwartz SGS100A RF source was used).

Supp. Fig. 1c shows the full block diagram of the measurement setup, including the attenuation of the RF lines, amplifiers, filters.

Supp. Fig. 1d shows the block diagram of the RF readout setup. The 3.95 GHz readout tone was generated by a Rohde&Schwartz SGS100A RF source, transmitted through the device and measured after down mixing a second signal using an IQ mixer (Marki Microwave MLIQ-0218L) and finally digitized and measured by the ZI UHFLI (shown by LI).

We performed a finite element 3D electrostatic simulation of device chip using Ansys Electronics to determine the capacitances between the gate and the wire, yielding about 30 aF and between the leads, listed in Supp. Table I. To estimate the effective capacitance, C_{eff} using the simulated values two things should be considered. First, the RF signal applied on the gate couples to both sides of the wire, hence the actual driving force of the displacement current is determined by the linear combination of four capacitances, $C_{\mathrm{tot}} = C_{\mathrm{G,I1}} + C_{\mathrm{G,V1}} - C_{\mathrm{G,I2}} - C_{\mathrm{G,V2}} \approx 54$ fF. Second, the excess charge in the given biased lead, induced by the RF signal could flow through the wire or from the bias tee. This will reduce the measured effective capacitance, C_{eff} compared to C_{tot}.

* csonka.szabolcs@ttk.bme.hu

† makk.peter@ttk.bme.hu

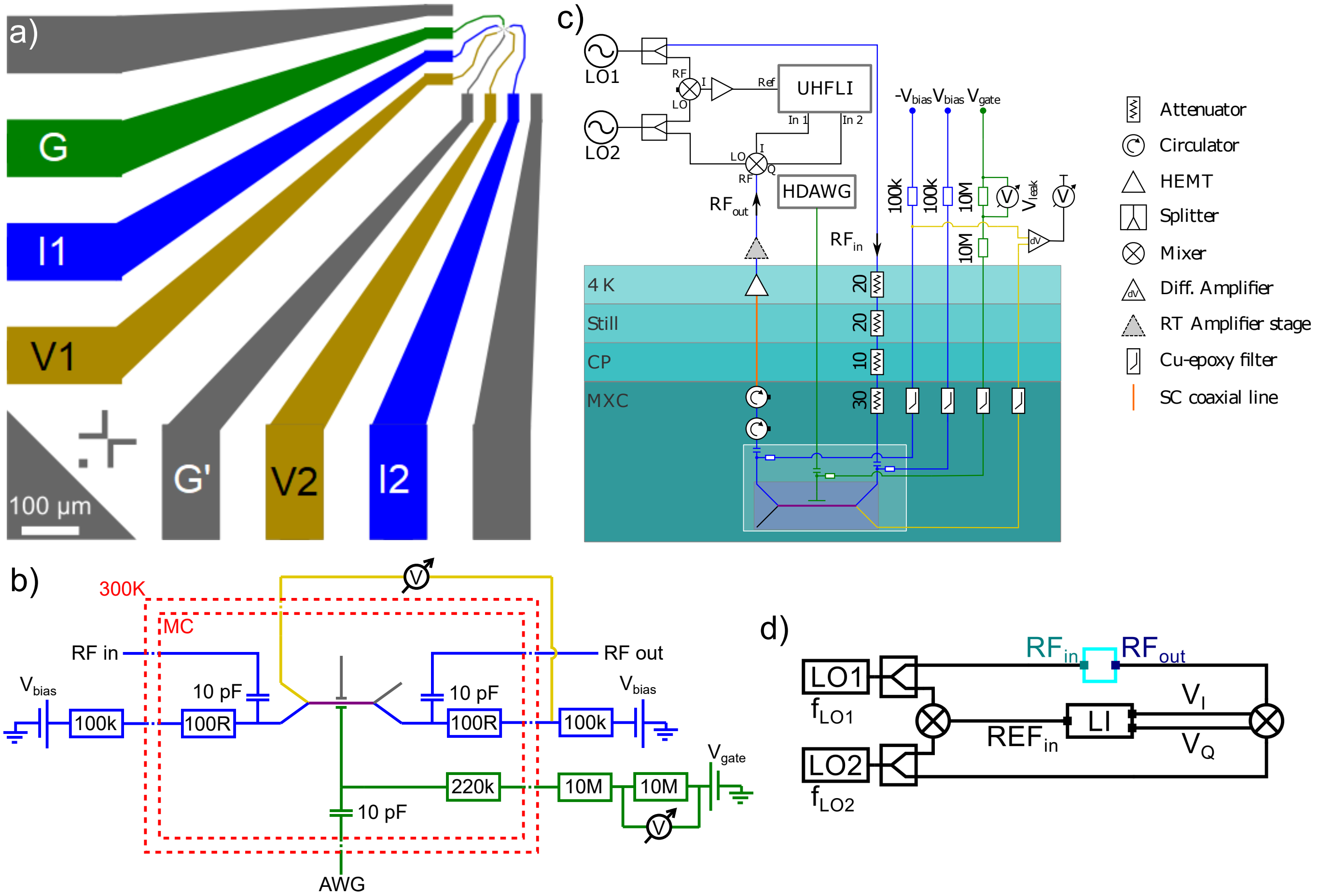

Supplementary Figure 1. Sample geometry and the measurement circuit. a) The chip design, indicating the contacts. b) Circuit diagram of the setup focusing on the on-board bias-tees and room temperature resistors. c) Complete measurement setup. d) Block diagram of the RF measurement setup, the probe signal was synthesized a local oscillator (LO1), using $f_{LO1} = 3.95$ GHz, downconverted by f_{LO2}, using IQ mixers and measured by the Zurich Instruments UHFLI.

	Gate	I1	V1	Gate'	V2	I2
Gate	93 fF	51 fF	18 fF	10 fF	7 fF	8 fF
I1	51 fF	128 fF	52 fF	12 fF	7 fF	7 fF
V1	18 fF	52 fF	125 fF	33 fF	12 fF	10 fF
Gate'	10 fF	12 fF	33 fF	126 fF	53 fF	18 fF
V2	7 fF	7 fF	12 fF	53 fF	132 fF	53 fF
I2	8 fF	7 fF	10 fF	18 fF	53 fF	96 fF

Supplementary Table I. Capacitance values between the leads calculated by electrostatic finite element simulation.

II. MICROWAVE TRANSMISSION OF THE NANOWIRE

First we compare the microwave transmissions when the wire is in the superconducting and the normal state. Here the superconductivity is quenched by passing 2.5 µA DC current, larger than the critical currents, through the wire. The obtained spectra are plotted in Supp. Fig. 2. The low frequency cut-off around 3.5 GHz is due to the bandwidth of our 4 K amplifier. There is clear difference in the transmission between the superconducting (blue) and the normal (orange) case. In general, the magnitude of the transmission is significantly suppressed in the normal state and the resonances originate from standing wave pattern in our setup. The red line indicates the chosen readout frequency of 3.95 GHz, where both the normal and the superconducting transmission and their difference is maximal.

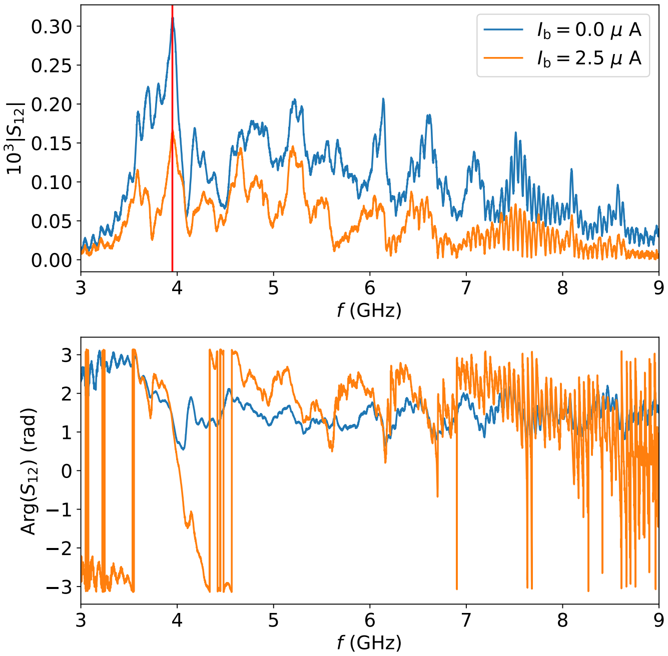

Supplementary Figure 2. Complex microwave transmission of the wire in superconducting (blue) and in normal state (orange). Red line marks the chosen 3.95 GHz readout frequency.

III. RELATING THE DC AND HETERODYNE MEASUREMENTS

In the main text we showed (see Fig. 2) that microwave transmission of the wire resembles well the features of the DC resistance measurement. Here we repeat the voltage drop and the magnitude of the heterodyne voltage plots (Supp. Fig. 3a and b, respectively) along with the differential resistance (panel c) and the phase (panel d).

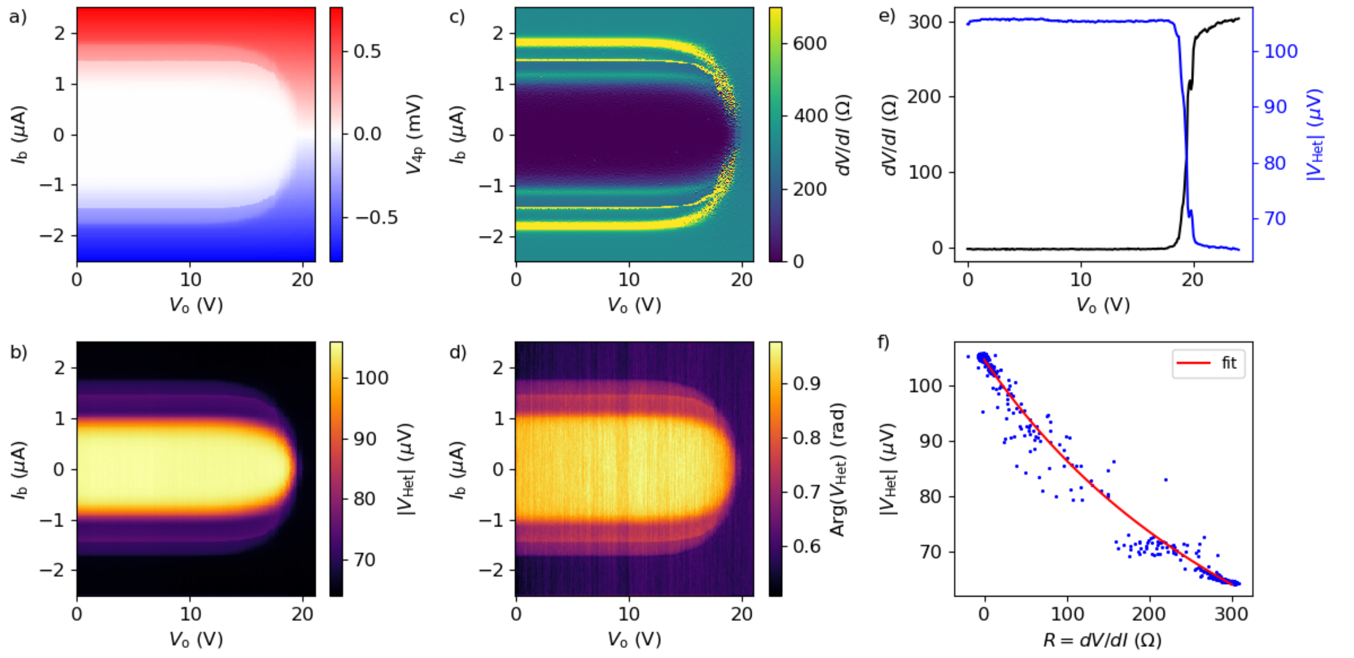

Supplementary Figure 3. Relation of the DC and RF measurement. Panel a-d) present the simultaneously carried out DC and RF characterization as the function of the bias current, I_b and the DC gate voltage, V_o. Panel a) shows the four-point voltage, V_{4p} (same as Fig. 2a of the main text), panel b) the calculated differential resistance, panel c) is the normalized magnitude of the transmission (same as Fig. 2b of the main text) and panel d) is the corresponding phase. Panel e) further illustrates the correlation of the RF transmission and the differential resistance. The curve are obtained by averaging the data of panels b and c for bias values $|I_{bias}| < 100$ nA. Panel f) demonstrates the phenomenological relation of the transmission and differential resistance. Points are taken from panels b) and c) for low bias values, below 100 nA.

To quantify the relation between the magnitude of the RF measurement and the resistance, these two quantities are plotted on panel e) and f) for the low bias region. In panel e) the data from panels b) and c) averaged for bias for $|I_b| < 100$ nA and the magnitude of the RF signal and the differential resistance is plotted as the function of the gate voltage. Similarly to Fig. 2d of the main text we observe a step-like decrease in the transmission when the resistance increases. To obtain a quantitative relation between the RF signal and the resistance panel f) shows the former as the function of the latter for bias values $|I_{bias}| < 100$ nA. Based on the transmission line theory one expects that

$$S_{12} = \frac{2Z_0}{2Z_0 + Z} \tag{S1}$$

for our geometry, where a lumped element Z impedance is placed in the way of the signal and $Z_0 = 50\ \Omega$ is the characteristic impedance of the coax cables [1]. Although, due to the complex RF environment (standing waves caused by reflections), this formula does not describe our data, we could use this functional shape to construct a phenomenological description of our data. This is plotted on panel f) with a red line as

$$|V_{\text{Het}}| = 104\ \mu\text{V} \cdot \frac{474\ \Omega}{474\ \Omega + R}, \tag{S2}$$

where $R = dV/dI$ is the differential resistance and the prefactor of 104 μV is the measured signal magnitude at zero gate and low bias. We emphasize that no conclusion is drawn based on this phenomenological formula, only it will be used to get a better comparison of our simulation and the experiment (see Methods of the main text).

IV. ADDITIONAL DATA ON TIME-RESOLVED PULSED MEASUREMENTS

In Fig. 3 of the main text it was shown that fast pulses generate a displacement current in the device and I_{disp} could switch the device to the normal state for the rising and the falling edge of the pulse. Here we repeat the measurement sequence of panel b), but with improved time resolution, using technique B, the main findings are summarized in Supp. Fig. 4. Panel a) shows the applied pulse sequence, a proper square pulse with 2.5 V amplitude. Panel b) shows the phase shift of the measured homodyne voltage at the function of time and the offset gate voltage, V_{o}. Similarly to Fig. 3b of the main text the threshold voltage shifts with $-V_{\mathrm{p}}$ and purple regions appear at both the rising and the falling edge of the pulse extending from the threshold down to zero voltage.

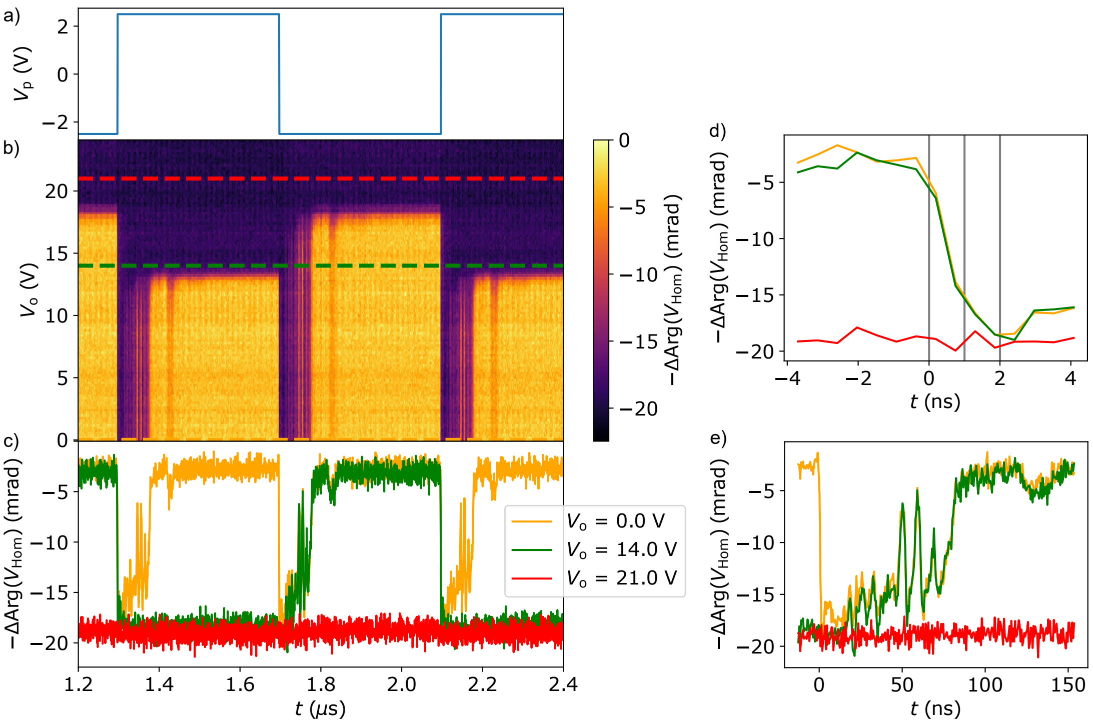

Supplementary Figure 4. Device response under fast pulses. a) the applied square wave with 2.5 V amplitude. b) the phase of the measured homodyne voltage as the function of the time and the offset gate voltage, measured with technique B. c) Three color coded time trace of panel b), d) a zoom-in to the rising edge of the pulse, showing a fast ≈ 2 ns switching time, e) a zoom in to the falling edge of the pulse highlighting the well-reproducing nature of the oscillations during the relaxation.

Three color coded horizontal cuts are shown on panel c) to the further highlight the main features of the measurement, taken at $V_{\mathrm{o}} = 0$ V (orange), at 14 V (green), close to the threshold and at 21 V, above the threshold (red). It is visible, that for zero gate voltage, the signal jumps each time the gate voltage changes, in agreement with the DCIS picture. The improved time resolution reveals that the signal relaxes on a timescale of 80 ns, with well-reproducing oscillations (compare the orange and green curves on panel e), a zoom-in to one of the falling edge of the pulse) observed during the relaxation. Also the well-reproducing nature is visible in the full gate dependence on panel b) as vertical features inside the purple regions. The ripples are absent when the DC gate voltage is high enough to keep the wire in normal state as shown by the red curve. The lack of the ripples exclude the possibility that they are simply the result of a crosstalk between the gate and the readout line, they only appear when the state of the wire changes. Due to the well-reproducing nature we suspect the relaxation feature is the result of multiple reflections of the gate pulse in the gate line (due to impedance mismatch) rather than the internal relaxation of the wire (see Sec. X for the detailed discussion).

Zooming in to the rising edge of the signal (corresponding to the switching from superconducting to normal state, shown on panel d) shows that the signal reaches the normal state value in less than 2 ns (comparable to the values obtained in the measurement with harmonic drive, see Fig. 6 of the main text and Sec. XI), which is one order of magnitude faster than the time scale presented in Ref. 2, demonstrating that the DCIS allows fast switching of the device to the normal state. This 1–2 ns is just an upper bound for the superconducting to normal switching time, since the measured timescale is at our instrumental resolution limit.

V. EVALUATING THE DISPLACEMENT CURRENT FROM RAMPED PULSES

In this section we elaborate on the experiments condensed into Fig. 3d of the main text. To investigate the effect of the displacement current, we applied trapezoidal shaped pulses on the gate and studied how the ramp time and amplitude of the pulse influences the time-resolved RF transmission of the device. The pulses were generated by a ZI HDAWG, and ranged in amplitude from 0.1 V to 5 V. Supp. Fig. 5a shows two examples for such measurements with fixed 2.5 V and 5 V pulse amplitudes with varied ramp time. Each row of the figure is an average of 2000 measurements using the same pulse sequence. In general, one can see on the figure a triangular purple region of normal state, that expands for longer ramp times. On the bottom of the figure, for the fastest ramping times, the signal resembled a step function: an abrupt change is visible from the SC state (yellow) to the normal state (dark purple). For longer ramp times the normal state region expands which forms the triangular region in the middle of the figure. Within this regions there is a displacement current flowing through the wire. The phase shift shows a monotonic decay towards longer ramp times and also exhibit stripe-like features parallel to the edge of the triangles. At the bottom third of the figure, where the signal changes quite rapidly, we see features to the right of the triangle, where no displacement current should flow. We consider that both of these effects are caused by reflections in the gate line as we discuss in Sec. IV and X.

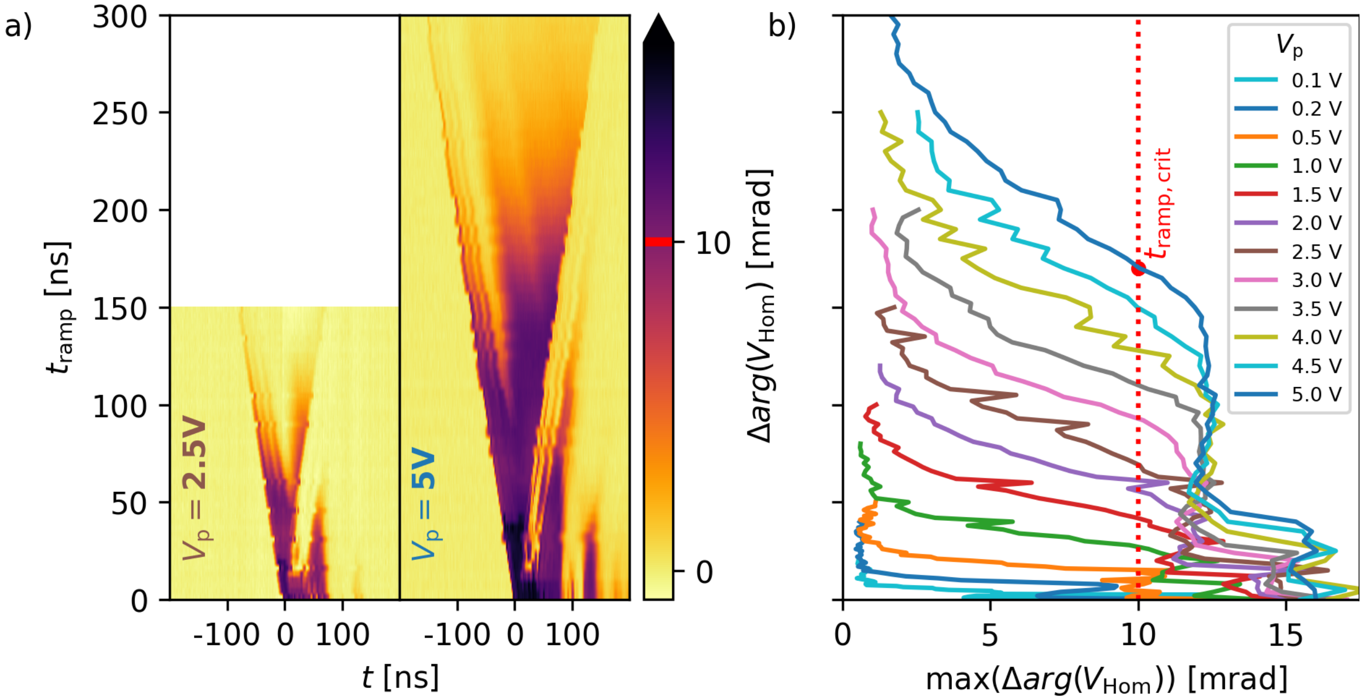

Supplementary Figure 5. Investigating the effect of ramping speed on the DCIS. a) Two examples for the timeresolved device response (phase shift of the transmitted RF signal) for trapeze-like, ramped pulses with varying ramp times and fixed amplitudes of 2.5 V (left) and 5 V (right) measured at $V_{\rm o} = 0$ V and $I_{\rm b} = 0$ A. The triangular region in the middle of the figure, are the point where the gate was actively ramped, thus resulting in a displacement current. The features right of the triangular region are due to reflections within the system. b) The maximal phase shift during each pulse sequence for twelve different amplitudes, the top blue and brown curves are generated from panel a). For longer ramp times, and thus lower displacement currents, the phase shift is smaller. Red line marks the threshold of critical ramp time, these values are plotted in Fig. 3d of the main text.

We can extract the maximal phase shift of the RF signal for each ramp time, as shown in Supp. Fig. 5b. The same experiment is repeated with smaller pulse amplitudes down to 0.1 V, and the extracted maximal phase shifts as the function of the ramp time are plotted as well. We can identify here three different regions. For the largest phase shift, about 15 mrad the device is fully in the normal state, hence the displacement current is larger than the largest switching current, about 1.8 µA. Below that there is a plateau at 12 mrad, where only part of the device is in the normal state, corresponding to the N' region, between $I_{\rm c1}$ and $I_{\rm c2}$. For longer ramp times the plateau ends with a kink, which corresponds to the lowest critical current, $I_{\rm c1}$. This is followed by a monotonous decay which we associate with a still superconducting state, but with an increased inductance due to a finite supercurrent flowing through the

wire.

We choose a threshold value of the phase shift 10 mrad, just below the kink, marked by the red line, to define the critical ramp time, $t_{\mathrm{ramp,crit}}$, separating the superconducting and the normal regions. With this value, we obtain $t_{\mathrm{crit,ramp}} \approx 170$ ns for 5 V amplitude. By repeating the same for each curve of panel b, we get the data shown in Fig. 3d of the main text. If due to some noise a curve crosses the red line multiple times, only the crossing at the shortest time is taken into account.

Using the value of the lowest critical current, the slope can be used to give an estimate for the effective capacitance, C_{eff}, through $I_{\mathrm{c1}} = 1.1$ µA $= I_{\mathrm{disp}} = C_{\mathrm{eff}} \Delta V_{\mathrm{g}} / \Delta t$, yielding 20 fF, which is in good agreement with the values of Supp. Table I.

Remark: the ramp time corresponding to the kink on panel b (i.e. I_{c1}) is about 3–4 times larger than the one at the step (i.e. I_{c2}), while the ratio of the critical currents is only $I_{\mathrm{c2}}/I_{\mathrm{c1}} \approx 1.5$. This means that a larger drive is needed to further increase the displacement current above I_{c1} than below.

VI. ADDITIONAL DATA ON SINE-DRIVEN MEASUREMENTS

The measurements here were performed on the same device but in a separate cooldown. The lowest critical current was about 25 % larger, presumably due to better thermalization and lower temperature, but the larger ones stayed the same. The figure shows three examples for the magnitude of the RF signal (measured by technique A, left column) and the differential resistance (right column) in case of continuously driven gate with a harmonic signal as the function of the driving amplitude, A_g and the DC bias current, I_b (similar to one shown in Fig. 4c of the main text). At low driving frequencies, $f_\mathrm{g} < 50$ MHz (see panel a for an example with $f_\mathrm{g} = 18$ MHz) the features are the same as in the main text. The fully superconducting region is restricted to a triangle at low driving amplitudes and currents. At zero bias for increasing amplitudes the transmission decreases in a step-like fashion as the induced displacement current reaches the switching currents. Most interestingly at high driving amplitudes, increasing the bias current from zero better transmitting regions (diagonal branches) can be reached, at these regions the DC current partially compensates the displacement current. The main features are well reproduced by the model described in the Methods of the main text and shown in Fig. 4d of the main text.

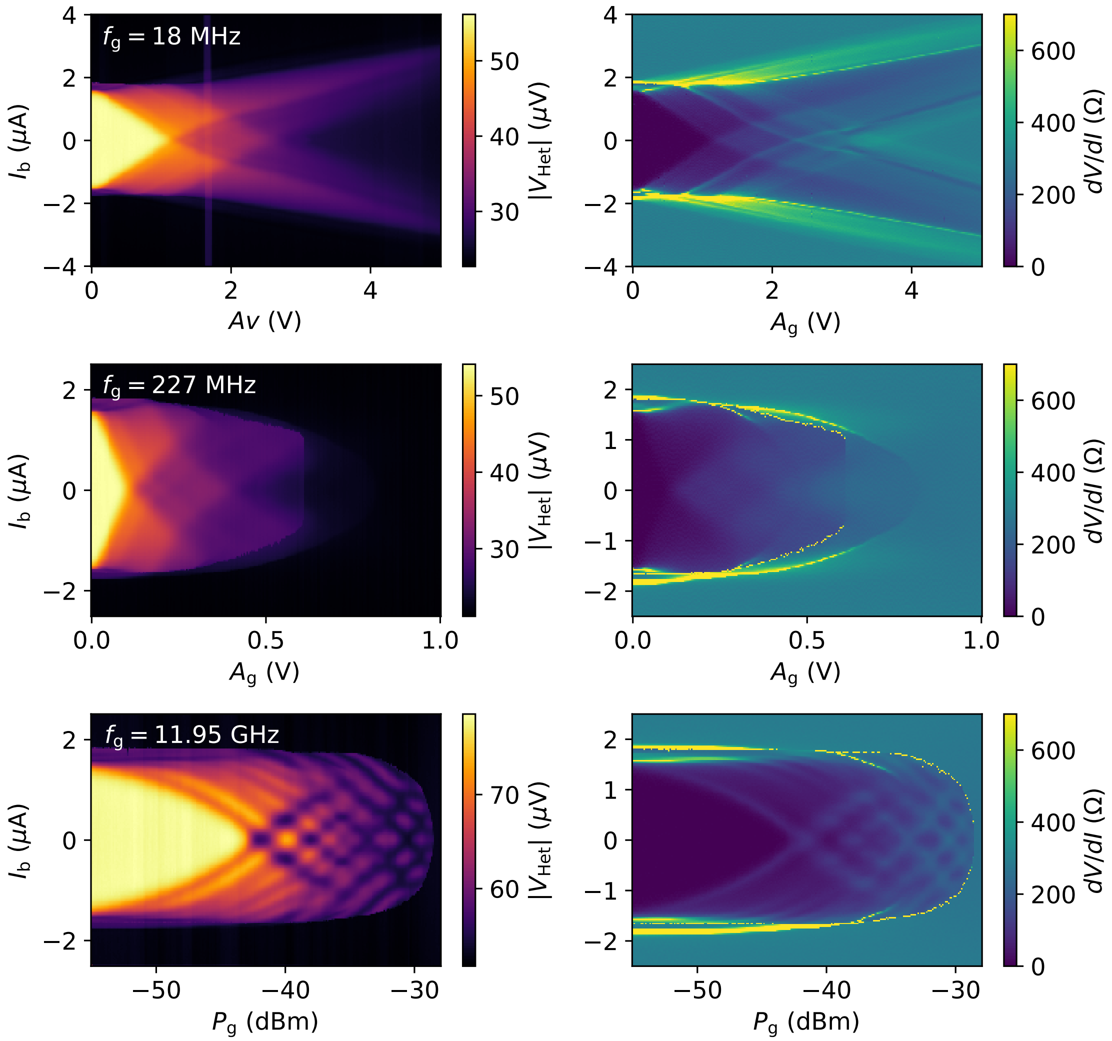

Supplementary Figure 6. Several example of continuously driven measurements from the second cooldown as the function of the driving amplitude, A_g and the DC bias current, I_b. Left column: the heterodyne signal, right column: the differential resistance at three different driving frequencies. a) At $f_\mathrm{g} = 18$ MHz the fully superconducting region is confined in a triangle at low driving amplitude and low bias. At larger amplitudes the displacement current can be partially compensated with a DC bias, producing the diagonal brighter regions. b) At $f_\mathrm{g} = 227$ MHz the diagonal features gets blurred, the non-fully normal region is confined into an elliptic region. c) At $f_\mathrm{g} = 11.95$ GHz Shapiro steps form inside the ellipsoid.

For higher frequencies the fully superconducting triangle is preserved but the diagonal branches at higher bias currents fade away, they are barely visible at $f_g = 227$ MHz in Supp. Fig. 6b. Meanwhile the partially superconducting region gets to confined to a half ellipse, the critical currents quadratically decrease with increasing gate amplitude instead of linearly. At even higher frequencies, above a few GHz, Shapiro steps develop in the partially superconducting region, see an example of $f_g = 11.95$ GHz in Supp. Fig. 6c.

In the main text we discussed that the tip of the triangle of the fully superconducting region also provides a way to determine C_{eff}. At zero bias, this is the point where the maximum of the displacement current reaches the first critical current, I_{c1}. In Supp. Fig. 7 we present the magnitude of the RF signal, $|V_{\text{Het}}|$ measured at zero bias as the function of the driving amplitude and frequency. This measurement was done in the first cool down, i.e. same as the main text. The border of the well transmitting (yellow) region is expected to be a hyperbola based on the displacement current mechanism (since $A \cdot \omega \propto I_c$). The overall trend follows the expectations with some slight deviations due to imperfections and standing waves in the gate line. The plotted line is the expected boundary using the previously obtained (see Sec. V) $C_{\text{eff}} = 20$ fF and $I_{c1} = 1.1$ µA, providing further experimental support that 20 fF is the value of the capacitive coupling.

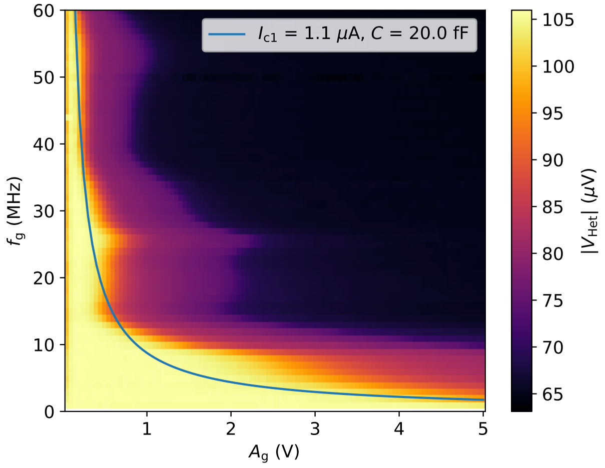

Supplementary Figure 7. Zero bias transmission under continuous harmonic driving. The measured heterodyne voltage as the function of the driving amplitude and frequency. Yellow marks the superconducting region. The blue line is the expected boundary of the superconducting region, where $\max(I_{\text{disp}}) = I_{c1} = 1.1$ µA, with $C = 20$ fF capacitance.

VII. ADDITIONAL FFT MEASUREMENTS

In the main text (Fig. 4a and b) we have shown that GCS effect allows for mixing the gate and the readout signal. The two examples with $f_g = 10$ MHz showed that at small amplitudes only the leakage-based gating effect is responsible for the mixing, producing sideband peaks at $f_{readout} \pm f_g$ only close to the threshold gate voltage. While for higher amplitudes the second sideband peaks at $f_{readout} \pm 2f_g$ also appear, and they extend down to zero offset gate voltage. We attributed them to the DCIS effect, which switches the wire to normal state twice in every period of the gate signal.

Here we show few more examples with different f_g driving frequencies and investigate the dependence of the FFT peak amplitudes on the DC gate voltage. Supp. Fig. 8a and b show the first and second sideband peaks, respectively, for four different f_g frequencies. All these measurements were carried out at the same 0.05 V rms signal level. Measurement with different amplitudes and frequencies were also carried out, yielding similar trends and conclusions as discussed below. The first sideband peak was only observed close to the threshold gate voltage and at low driving frequencies, up to $f_g \approx 40$ MHz (see panel a). The second side band peak (panel b) can exhibit different trends with the DC offset gate voltage at different frequencies. At the lowest frequency of 30 MHz (blue curve) only a small peak appear at the same voltage range as the first sideband peak, around 13 V. Presumably this is only the naturally present higher harmonic of the gating peak and does not have different origin. For higher frequencies the displacement current becomes non-negligible, significant second sideband peaks develop without any measurable first ones. At 60 MHz (orange curve) the peak is only present close to the threshold, between 9 and 12 V. Towards even higher frequencies, the peak extends down to 0 V (see the green and red curves for 75 and 90 MHz, respectively). The difference between these two is that for the green curve the peak increases close to the threshold and decreases at higher gate voltages, but for the red one it starts from its maximal value and it only decays. Note that threshold voltage slightly decreased with time due to the training effect (see the Methods of the main text).

In the next section we present a simple model, that generates the curves in Supp. Fig. 8c and d, that captures the main features discussed above.

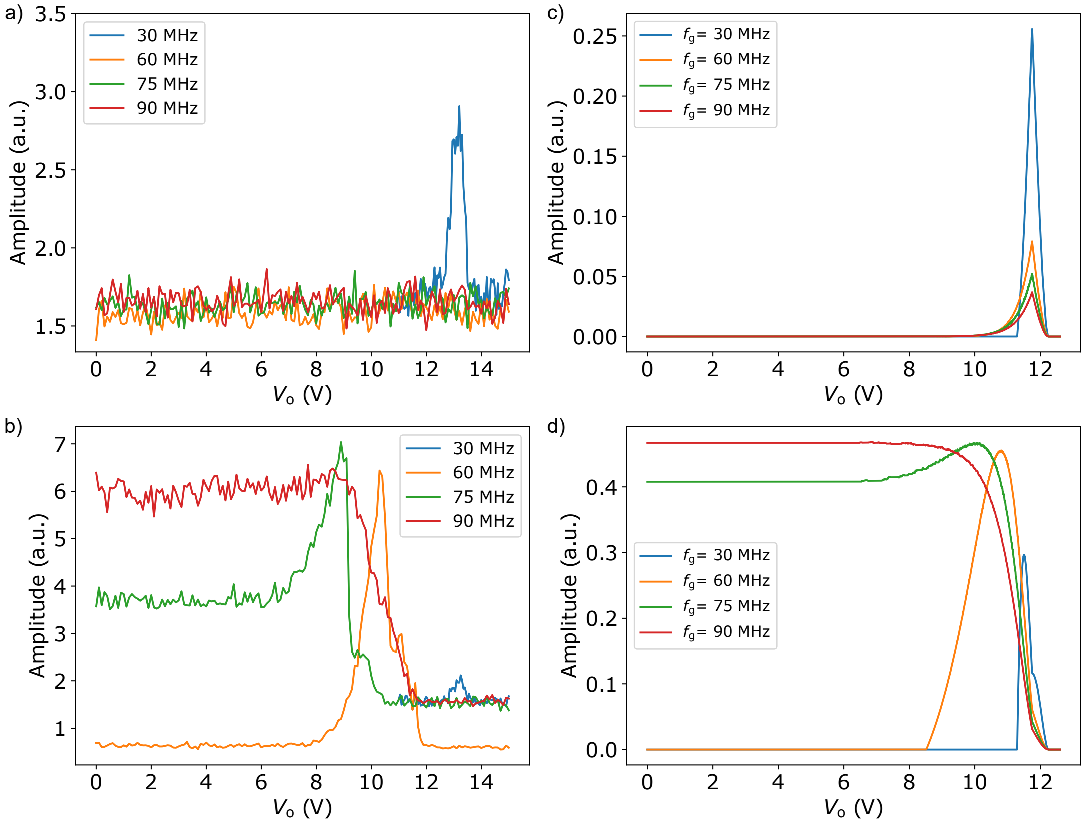

Supplementary Figure 8. Gate dependent FFT curves at different f_g drive frequencies. a,b) measurement with fixed 50 mV rms signal level, c,d) simulation, a,c) first sideband peaks, b,d) second sideband peak

VIII. MODELING THE FREQUENCY DOMAIN MEASUREMENTS

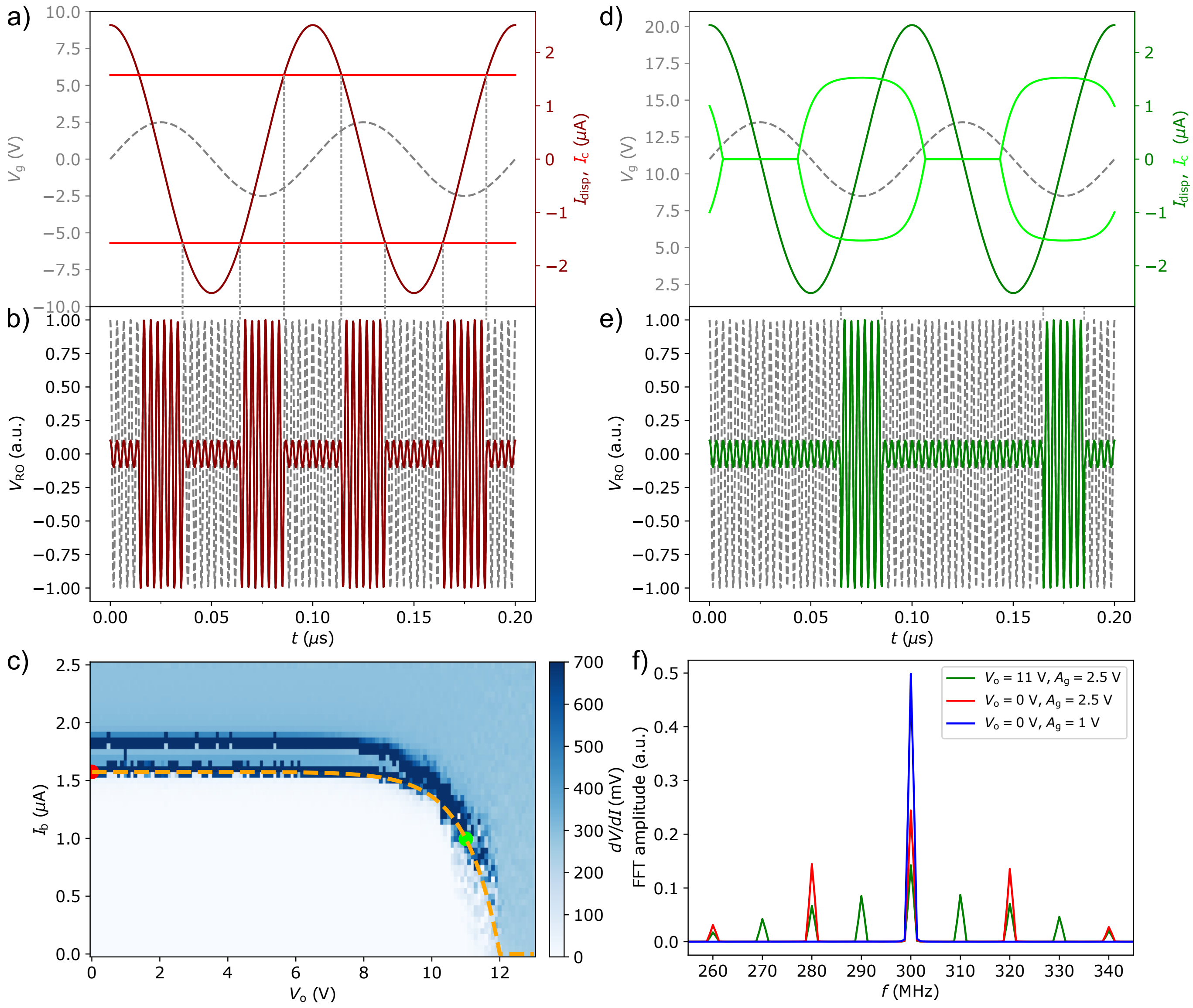

Supplementary Figure 9. Modeling the frequency domain measurements. a) The AC signal on the gate (10 MHz with 2.5 V amplitude, gray dashed line) induces a displacement current in the wire (brown line), which is compared to the critical current (red lines). b) The incoming (gray dashed line) and transmitted readout signal (brown) is modeled with a 300 MHz tone, and the transmission drops from 1 to 0.1 when the wire is driven normal by the displacement current. c) A GCS measurement as the derivative of the voltage drop, and overlaying the $I_c(V_o)$ curve (Eq. (S3)) with yellow dashed line that is used in the calculations. d,e) same as a) and b) at $V_o = 11$ V, where the switching current (lime curve) is modulated AC signal on the gate. f) three calculated FFT spectra, red and green one corresponding to the cases illustrated on panels a-b) and c-d), exhibiting 2 and 4 pair of sideband peaks. The blue one is a reference curve calculated at $V_o = 0$ V with $A_g = 1$ V amplitude such that the wire stays in the SC state, hence there are no sideband peaks due to the modulation.

Here we outline a simple model to understand the features of the frequency domain measurements presented in the previous section. First, let us focus on the case when only the DCIS matters, i.e. at low DC gate voltages. Supp. Fig. 9a illustrates the applied gate signal (gray dashed line with $f_g = 10$ MHz and 2.5 V amplitude), the induced displacement current (brown line) and the critical current (red lines), I_c and $-I_c$. When the displacement current exceeds I_c, the wire switches to normal state. Panel b illustrates the readout signal, modulated by the state of the wire. The gray dashed line is the applied readout signal, while the brown one is the transmitted one. For

computational reasons the readout signal is modeled with $f = 300$ MHz, and when the wire is in the normal state, the transmission and hence the amplitude of the transmitted signal is simply reduced by a factor of 10. Finally the signal is analyzed by a FFT and the obtained spectrum is plotted on panel f with red line. The large, central peak at $f = 300$ MHz corresponds to the carrier signal, besides four additional peaks appear at 300 ± 20 MHz and 300 ± 40 MHz corresponding to the DCIS effect which switches the wire to normal state twice every period. As a comparison see the blue curve where the gate amplitude is small enough, 1 V, to avoid the switching of the wire, hence only the 300 MHz peak is present.

To take into account the gating effect in the presented model we allow for the change of the switching current with signal on the gate. The GCS characteristics is modeled by

$$I_\mathrm{c}\left(V_\mathrm{o}\right) = 1.575\ \mu\mathrm{A}\left[1 - \exp\left(\frac{V_\mathrm{o} - 12\ \mathrm{V}}{1\ \mathrm{V}}\right)\right]\Theta\left(12\ \mathrm{V} - V_\mathrm{o}\right), \tag{S3}$$

which is plotted in Supp. Fig. 9c on top of an actual DC GCS measurement, where the derivative of the voltage drop is plotted for clarity. Let us consider the $V_\mathrm{o} = 11$ V working point (lime marker), close to the threshold. Similarly to panel a, panel d shows gate signal (gray dashed line), the displacement current (green) and switching currents (lime). The main difference is that here the switching current is not constant, but changes with the AC signal on the gate according to Eq. (S3), even it is zero for a finite time close to the maxima of the AC signal. The readout signal (panel e) obtained similarly, the amplitude of the 300 MHz signal is reduced by a factor of 10, when the displacement current exceeds the actual critical current. Finally, the obtained FFT spectrum is shown of panel f with green curve. Here there are four pairs of peaks appearing around the central one with 10 MHz spacing. The odd harmonics originate form the gating effect, since it causes the switching once every period, when the gate signal is maximal.

We use the outlined method to qualitatively reproduce the offset gate voltage dependent FFT curves presented in Supp. Fig. 8a and b. The calculated curves are shown on panels c and d. The main tendencies of the offset voltage and the frequency dependence of the measurements are reasonably reproduced by our model giving a further support to the simultaneous presence of the two switching processes, the DCIS and leakage-related one.

IX. EVALUATING THE TIME SCALES OF THE LEAKAGE CURRENT BASED SWITCHING

Here we show the detailed steps, that lead to the results presented in Fig. 5 of the main text. Supp. Fig. 10b shows the whole measurement, the out-of-phase component (or Q quadrature) of the homodyne voltage, $\mathrm{Im}(V_{\mathrm{Hom}})$ as the function of the time and DC gate voltage, with 10 up/down pulses using 100 ns ramp time with 2.5 V amplitude. Parts of this measurement are also shown in Fig. 5. All measured points are used to construct the histogram shown in Supp. Fig. 10a, which shows distinct peaks for the superconducting and the normal state. Taking the values at one fifth of the peak heights, we define two thresholds, 3.13798 mV, below which the system is considered to be in the superconducting state (light blue), and 3.13803 mV, above which the system is completely in the normal state (red). Using these threshold values all the points of the measurement can be classified, which is illustrated on panels c) and d) for two periods. We focus on the border of the red and white regions from each row, we consider these points where the switchings occur (white dots of Fig. 5 of the main text and Supp. Fig. 11). As we outlined in the main text the expected shift of the threshold voltage can be determined using three things: i) the threshold voltage is defined as the position of the horizontal red-white boundary before the pulse starts, ii) the pulse timing is determined by vertical, purple stripes in the superconducting region and iii) the amplitude and the ramp time of the pulse gives the shifting. The obtained curve is shown on panel c) with blue dashed line (same as the blue line in Fig. 5 of the main text). The time delays, used to construct the histogram in the right panel of Fig. 5 of the main text, are the horizontal difference of the expected threshold positions and the actual switchings.

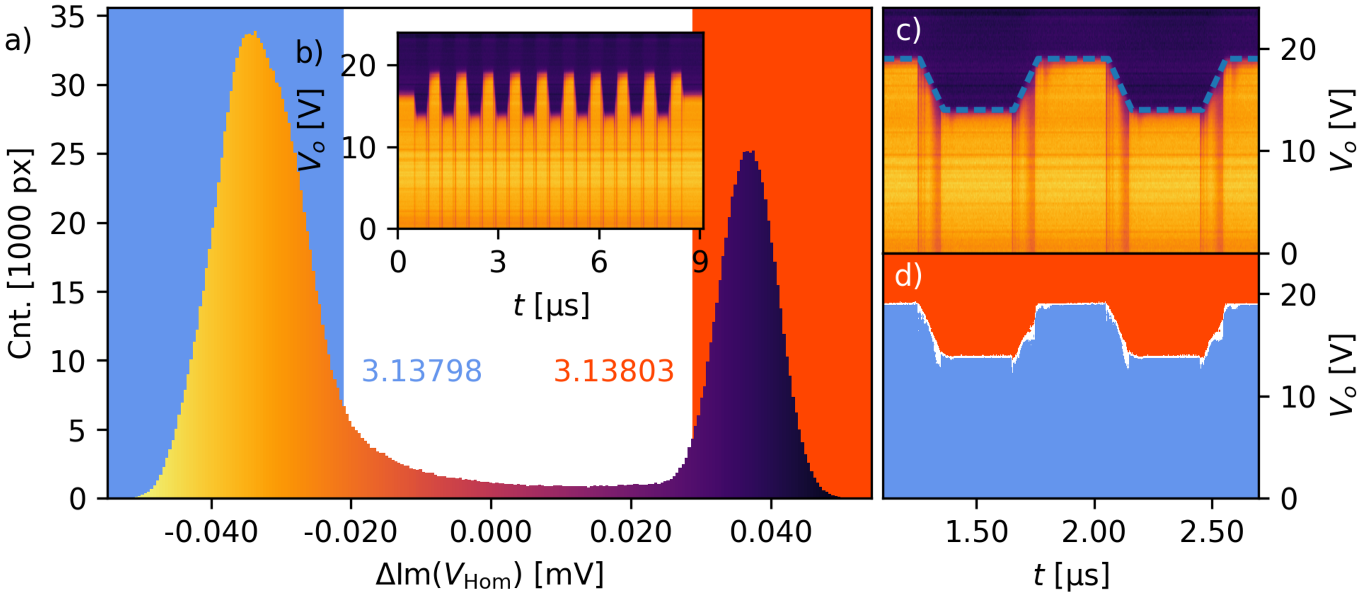

Supplementary Figure 10. Detailed analysis of the pulsed measurements. a,b) The out-of-phase component of the transmitted RF signal, as a function of time and DC gate voltage, with trapeze-like pulses (2.5 V amplitude, 100 ns ramp time) applied to the gate. The inset shows the whole measurement. From this measurement, we generate a histogram of all measured points. This shows two distinct peaks, corresponding to the fully superconducting and the fully normal state of the wire. We can assign threshold values at one fifth of the peak heights, thus we can separate three regions: superconducting (light blue), normal (red), intermediate (white). c) Zoom-in for two periods. The blue dashed line is the expected threshold position. d) Applying the thresholds calculated on panel a to panel c, to classify each point to three groups, blue for the superconducting state and red for the normal. Red-white boundary is defined as the position of the switchings.

Supp. Fig. 11 shows another example of the timescales of leakage-current-based switching. Here the pulse amplitude is similarly 2.5 V, but the ramp time is shorter, 60 ns. This is short enough that the displacement current exceeds I_{c1}, but not I_{c2}, hence part of the device is already driven normal during the ramping. We obtain similar timescales as for the longer ramp times, 2–3 ns delay for the N'→N switching and $\sim$ 15 ns delay for the N→N' transition.

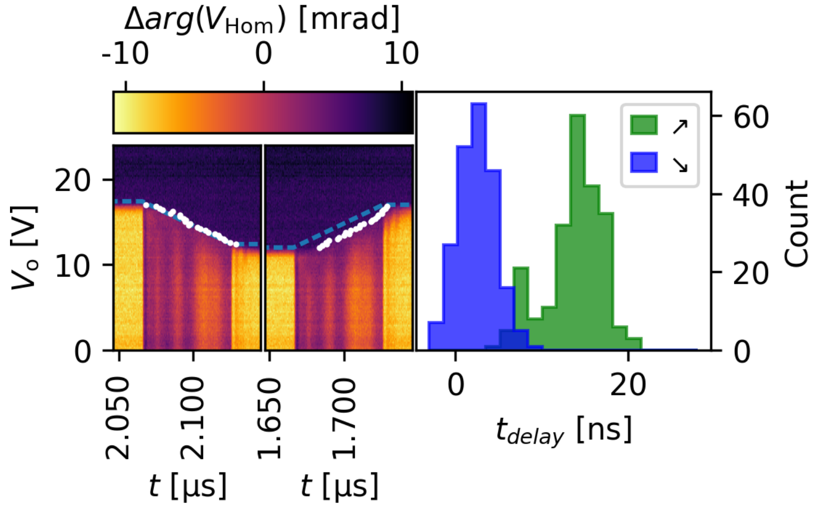

Supplementary Figure 11. Another example for the timescales of the leakage-current-driven operation. Here the pulse amplitude is similarly 2.5 V, but the ramp time is shorter than in the main text, 60 ns. The delays are 3–5 ns for the N'→N switching and ≈ 15 ns for the N→N' transition.

X. TIME-RESOLVED SINE-DRIVEN MEASUREMENTS

The measurements presented up to now could not distinguish between the cases when i) the gate dominantly couples to only one of the electrodes, and hence the displacement current flows uniformly in the wire; and ii) the gate couples to the middle of the wire and the displacement current flows asymmetrically in the halves of the wire. Using the model presented in the Methods of the main text and taking the wire response to a sinusoidal excitation in the time domain, one could distinguish between the two cases by adding and external DC current. Three of such simulations are presented in Supp. Fig. 12, where an 18 MHz gate signal is switched on for two periods. On panels a and b the AC current is homogenous in the wire, while on panel c it is opposite in the two halves of the wire. In the homogenous case the different regions just simply shift sinusoidally in the bias direction along with the gate drive. At each point of the plot the total current, i.e. $I_\mathrm{b} + I_\mathrm{disp}$ determines the actual state of the wire. On contrary, in the right panel regions with intermediate transmission appear close to the critical current. In these regions, at a given time, for half of the wire the displacement current compensates the DC bias, $I_\mathrm{b} + I_\mathrm{disp,1} < I_\mathrm{c1}$, while for the other half they add up $I_\mathrm{b} + I_\mathrm{disp,2} > I_\mathrm{c1}$ (with $I_\mathrm{disp,1} = -I_\mathrm{disp,2}$). Hence only half of the wire is in the superconducting state and other half is normal, leading to an intermediate transmission.

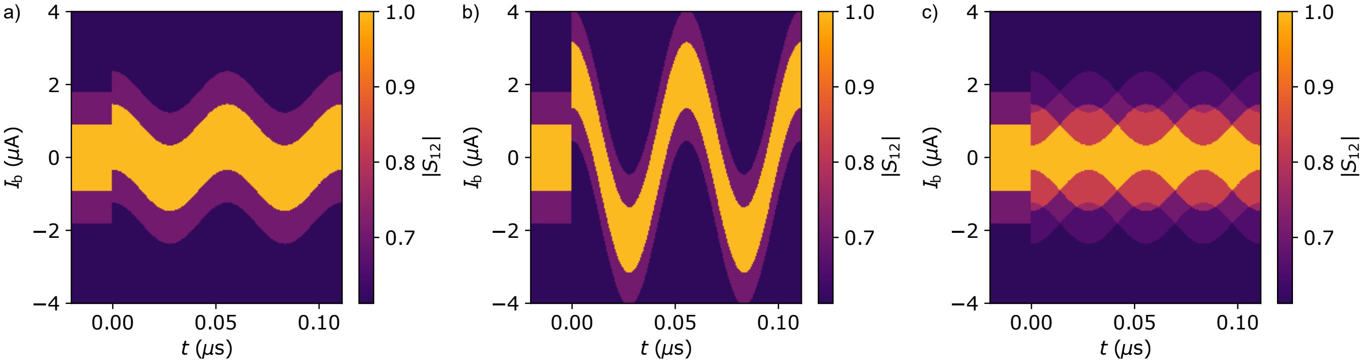

Supplementary Figure 12. Simulation of the time-resolved transmission for two different cases. a-b) the displacement current homogeneously flows in the wire with different drive amplitudes, c) it flows in the opposite direction in the two halves.

Such measurements were carried out at zero DC offset gate voltage and are presented in Supp. Fig. 13 for $f_\mathrm{g} =$ 18 MHz and five different amplitudes. Panel a) shows the time averaged transmission already presented and discussed in Sec. VI, the vertical lines mark the amplitude values used in the rest five panels. These show the time-resolved transmission (phase of the detected homodyne voltage, $\mathrm{Arg}\left(V_\mathrm{Hom}\right)$) zoomed in for the first roughly two and half period of the gate signal. Before switching on the gate pulse ($t < 20$ ns) at low DC bias currents the wire is in the superconducting state (yellow) while at high bias it is normal (purple). At roughly $t_0 = 20$ ns the gate voltage starts to increase as $\sin\left(\omega\left(t - t_0\right)\right)$, so the displacement current abruptly jumps from zero to its maximal value. After this point the yellow superconducting region shift roughly sinusoidally with the gate signal along the I_b direction. This feature well resembles Supp. Fig. 12a and b, supporting our claim that the displacement current flows homogeneously in the nanowire. The measurement shown in Fig. 6b of the main text is similar to the ones presented here, only focusing on the response after the transients decayed.

Interestingly the I_c2 boundary moves much less than the I_c1 with the AC drive, even, for the lowest amplitude, $A_\mathrm{g} = 0.5$ V on panel b) the shift is not resolved. This translates to the widening of N' state, the light purple region between I_c1 and I_c2. This is consistent with the previously discussed (see Sec. V) features that much larger driving is needed to reach the second switching, than to reach the first one. Presumably once the wire becomes resistive, that limits the displacement current through the wire.

The sudden jump of the displacement current is followed by several oscillation (this is more visible with intermediate amplitudes e.g. $A_\mathrm{g} = 1$ or 1.5 V on panel c and d, respectively). These oscillations can be qualitatively understood by a simple extension of the previous model. From transmission line point of view the gate electrode is an open end, so a perfect reflection occurs. However if there is an additional impedance mismatch along the cable, another reflection could occur there. This is illustrated in Supp. Fig. 14a, where the point of the impedance mismatch is indicated by a box, which could be described by an $\hat{S}$ scattering matrix, from which only the R reflection coefficient is interesting for us. The finite cable length between the point of mismatch and gate electrode translates to a time delay, Δt between the incoming and the reflected signal. After multiple reflections the total voltage on the gate reads as

$$V_\mathrm{g}\left(t\right) = A_\mathrm{g}\sin\left(\omega t\right)\Theta\left(t\right) + RA_\mathrm{g}\sin\left[\omega\left(t+\Delta t\right)\right]\Theta\left(t+\Delta t\right) + R^2 A_\mathrm{g}\sin\left[\omega\left(t+2\Delta t\right)\right]\Theta\left(t+2\Delta t\right) + ... \tag{S4}$$

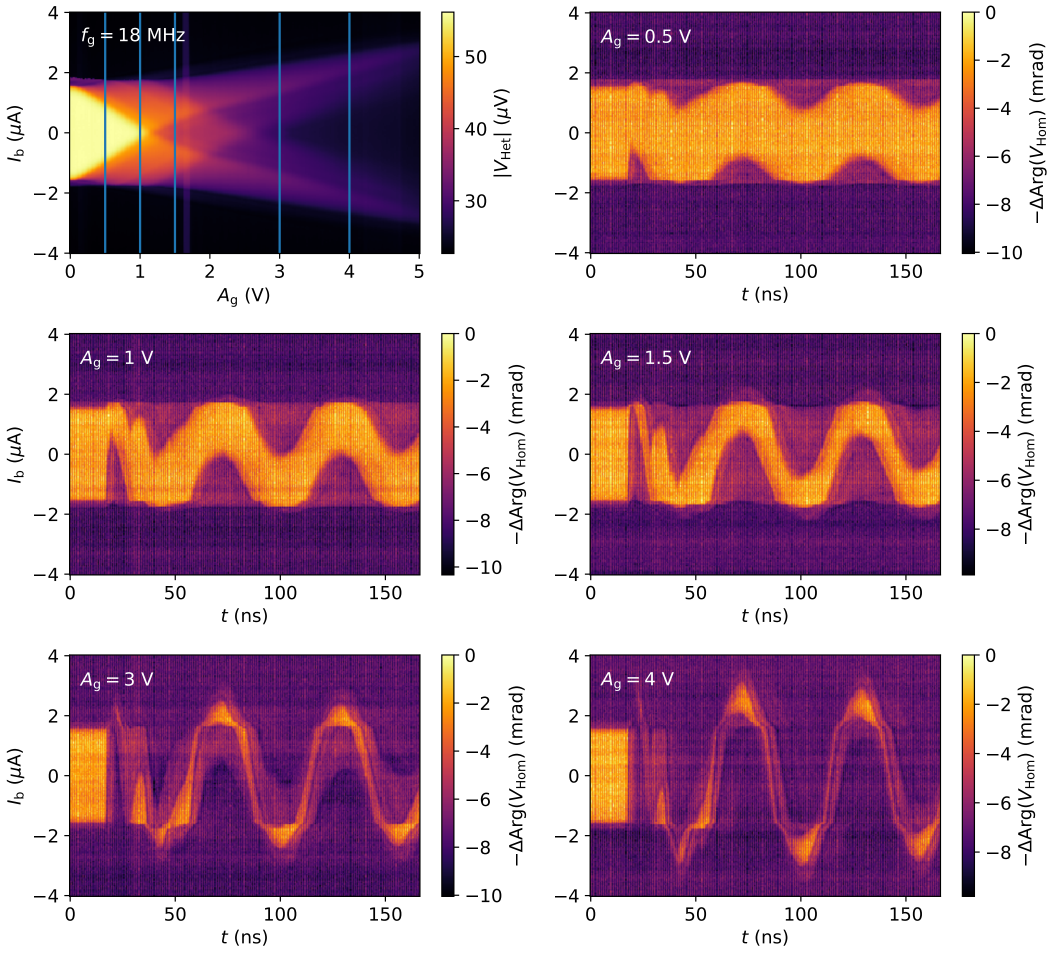

Supplementary Figure 13. Timeresolved transmission under harmonic drive. a) The averaged measurement, same as in Supp. Fig. 6, the vertical lines show at which amplitudes the rest five panels were measured.

Including this voltage dependence in our formalism generates Supp. Fig. 14c, where the sudden jump at $t = 0$ is followed by several smaller jumps with $\Delta t = 13$ ns spacing. Therefore, assuming a single point like defect could qualitatively capture the oscillations observed in the experiment (Supp. Fig. 14b, same measurement as Fig. 6b of the main text, but shown from the beginning of the pulse).

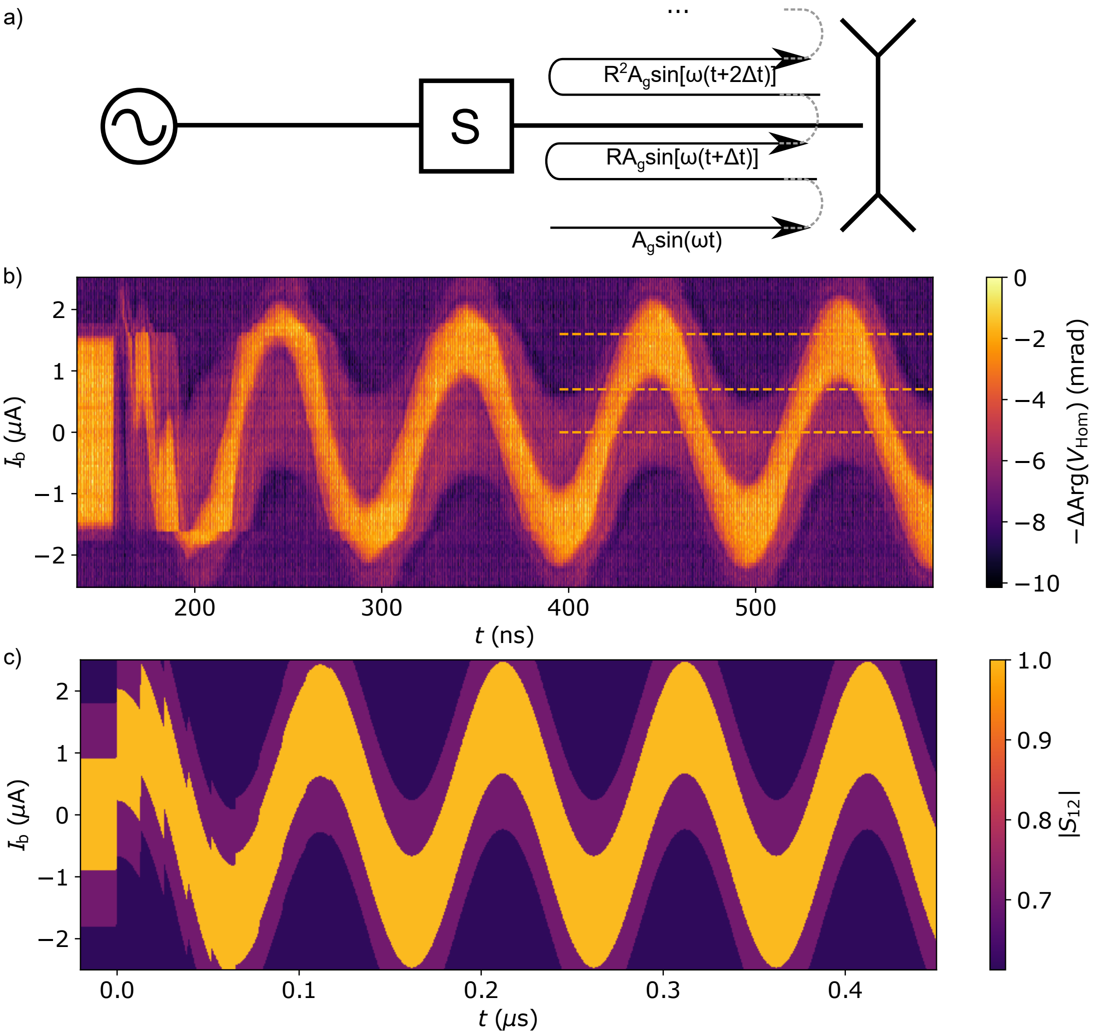

Supplementary Figure 14. Modeling the transient oscillation. a) schematics of the model. A point-like scattering center, S (e.g. impedance mismatch) and the *open end* nature of the gate electrode causes multiple reflection of the pulse in the gate line. b) Device transmission under harmonic drive with sudden start. Several oscillation distorts the first 1–2 period of the device response. c) Simulation of the device transmission taking into account the multiple reflections and qualitatively reproducing the oscillations.

XI. FURTHER DATA ON THE TIME SCALES OF DCIS

Here we show few more examples of fitting the switching times of the DCIS, similar to Fig. 6c of the main text. These are shown in Supp. Fig. 15 for $I_\mathrm{b} = 0, 0.7$ and 1.6 µA (along the dashed lines in Supp. Fig. 14). In the legend the timescales from top to bottom corresponds to fitted curves from left to right. Similarly to the main text we obtain 2.5–5.5 for the N'→S transition and 1.5–3 ns for the S→N' and N'→N switching times.

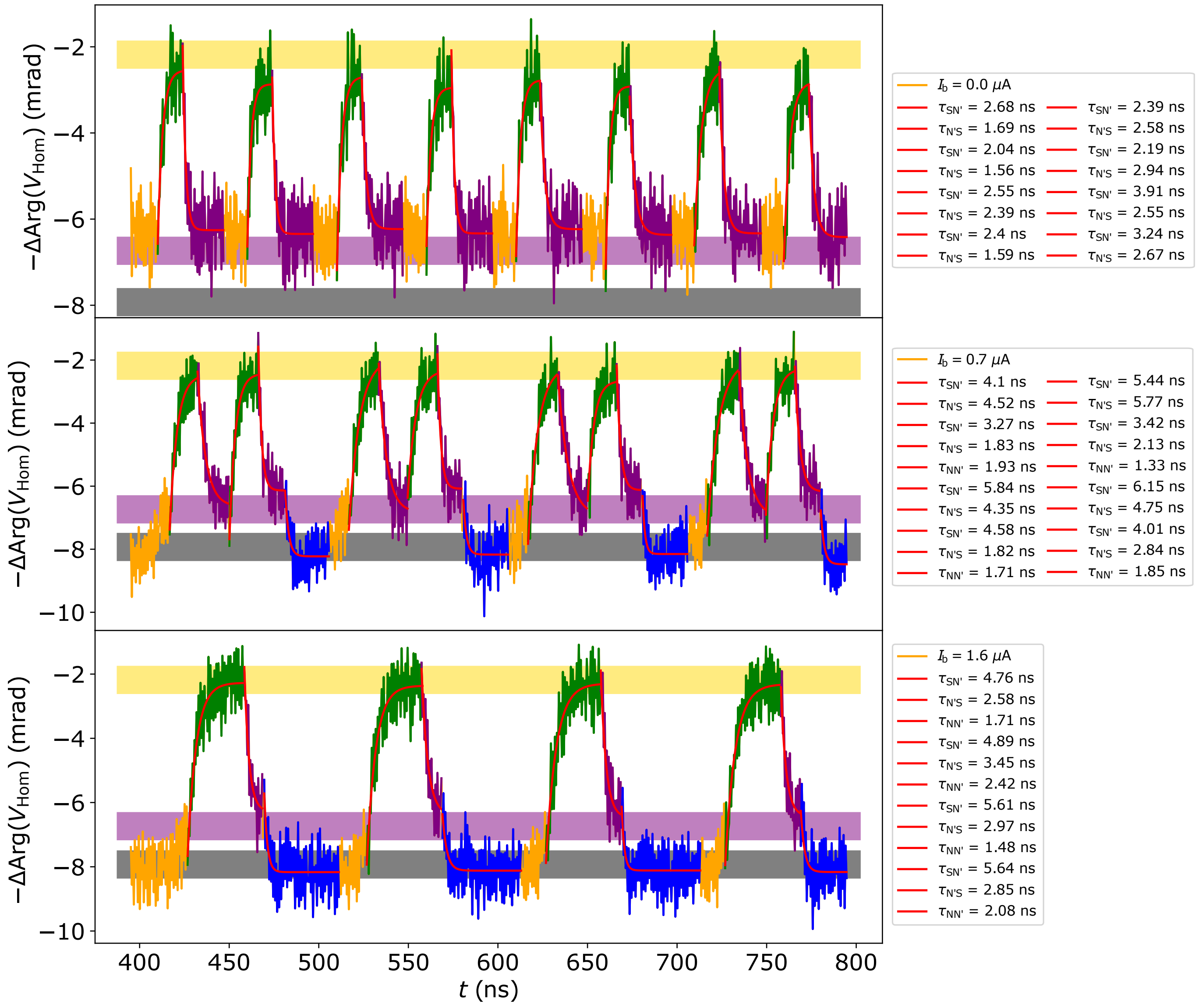

Supplementary Figure 15. Several more examples of the time scales corresponding to the DCIS.

Altogether we evaluated 100 N'→S, S→N' and 40 N'→N transitions at 17 different bias values (4 period of the signal per bias) to obtain the values of $\tau_{\mathrm{SN'}} = 3.9 \pm 1.3$ ns, $\tau_{\mathrm{N'S}} = 2.8 \pm 0.8$ and $\tau_{\mathrm{NN'}} = 1.4 \pm 0.5$ ns.

XII. ESTIMATES FOR POWER DISSIPATION

For an application one of the most important question is the power dissipated by an individual transistor. Depending on the given application the wire may be expected to stay in normal state only for finite time, e.g. in case of a microwave discriminator or for indefinitely, as for a memory-like application. Hence a permanent normal state operation gives an upper bound for the power dissipation. The first possibility is keeping the wire in normal state by DC bias current, in this case the power dissipation is

$$P = I_c^2 R \approx (1.8\ \mu\text{A})^2 \cdot 300\ \Omega \approx 1\ \text{nW}. \tag{S5}$$

Another possibility to keep the wire in normal state is by stationary leakage current, the dissipation is

$$P = I_{\text{leak}} V_{\text{th}} \approx 0.3\ \text{nA} \cdot 19\ \text{V} \approx 6\ \text{nW}, \tag{S6}$$

where the number were taken from Fig. 2 of the main text. Third way to drive the device stationary to the normal state is by applying a continuous drive to the gate. To determine the necessary power, we repeated the measurement shown on Supp. Fig. 7, but up to 12 GHz, measured in the second cooldown and is shown on Supp. Fig. 16. Again the yellow region is the fully superconducting state, whose boundary can be approximated as $CA\omega = I_{c1}$, and it is plotted by the blue curve, using $C = 20$ fF and $I_{c1} = 1.58$ µA. To reach the fully normal state, one need roughly four times larger drive amplitudes, shown by the orange curve and in accordance with the previous findings discussed in Supp. Info. V. To estimate the dissipated power we assume a series RC circuit formed by the gate-wire capacitance of $C_{\text{eff}} = 20$ fF and the $R = 300\ \Omega$ normal state resistance of the wire. Taking the drive amplitude given by the orange curve, the dissipated power is about 5 nW, almost independently of the drive frequency. The *working point* of the measurement presented in this paper were $I_{\text{bias}} = 0$ and $V_o = 0$, hence yielding an idle state dissipation of zero. An application may require a different working point, which may yield a finite dissipation in the idle state of the device. These number are comparable to ones on nTron devices [3].

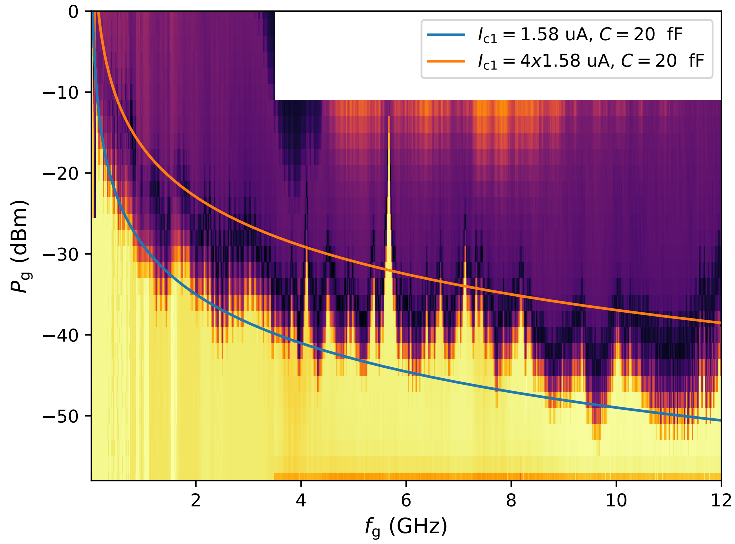

Supplementary Figure 16. Zero bias transmission under continuous harmonic driving. The measured heterodyne voltage as the function of the driving power and frequency. Yellow marks the fully superconducting region. The blue line is the expected boundary of the superconducting region, where $\max(I_{\text{disp}}) = I_{c1} = 1.58$ µA, with $C = 20$ fF capacitance. The orange curve is the boundary of the fully normal state at four times larger amplitudes than the blue curve.

Ref. 3 also addresses the question of jitter and finds 33 ps. Our instruments did not allow for such good time-resolution, but the measurements presented above allow us to give an upper bound for the jitter. As the time-resolved measurements are the average of 500-2000 independent measurements, any jitter would result in the broadening of the measured features. Supp. Fig. 4d shows a ~ 1 ns broad step for switching from S to N. The width of such step

originates from the combination of the intrinsic switching time, the jitter of the switching, the jitter of the instrument synchronization and the bandwidth of the instruments, giving an upper bound of 1 ns for the first three.

[1] D. M. Pozar, Microwave engineering (Fourth edition. Hoboken, NJ : Wiley, [2012] ©2012, [2012]) includes bibliographical references and index.

[2] F. Joint, K. R. Amin, I. Cools, and S. Gasparinetti, Dynamics of gate-controlled superconducting dayem bridges (2024), arXiv:2405.07377 [cond-mat.supr-con].

[3] K. Zheng, Q.-Y. Zhao, L.-D. Kong, S. Chen, H.-Y.-B. Lu, X.-C. Tu, L.-B. Zhang, X.-Q. Jia, J. Chen, L. Kang, and P.-H. Wu, Characterize the switching performance of a superconducting nanowire cryotron for reading superconducting nanowire single photon detectors, Scientific Reports **9**, 16345 (2019).
